# Aberrant Expression of SLC7A11 Impairs the Antimicrobial Activities of Macrophages in *Staphylococcus Aureus* Osteomyelitis in Mice

**DOI:** 10.7150/ijbs.93592

**Published:** 2024-04-22

**Authors:** Bingsheng Yang, Wen Shu, Jin Hu, Zhongwen Wang, Jichang Wu, Jianwen Su, Jianye Tan, Bin Yu, Xianrong Zhang

**Affiliations:** 1 Division of Orthopaedics and Traumatology, Department of Orthopaedics, Nanfang Hospital, Southern Medical University, Guangzhou, Guangdong Province, China.; 2 Guangdong Provincial Key Laboratory of Bone and Cartilage Regenerative Medicine, Nanfang Hospital, Southern Medical University, Guangzhou, Guangdong Province, China.; 3 Department of Trauma Orthopedics, Liuzhou People's Hospital, Liuzhou, Guangxi, China.; 4 Department of Orthopedics, The Second Affiliated Hospital of Nanchang University, Nanchang, Jiangxi, China.

**Keywords:** *Staphylococcus aureus*, Osteomyelitis, SLC7A11, Macrophage, Lipid peroxidation, PD-L1

## Abstract

*Staphylococcus aureus* (*S. aureus*) persistence in macrophages, potentially a reservoir for recurrence of chronic osteomyelitis, contributes to resistance and failure in treatment. As the mechanisms underlying survival of *S. aureus* in macrophages remain largely unknown, there has been no treatment approved. Here, in a mouse model of *S. aureus* osteomyelitis, we identified significantly up-regulated expression of SLC7A11 in both transcriptomes and translatomes of CD11b^+^F4/80^+^ macrophages, and validated a predominant distribution of SLC7A11 in F4/80^+^ cells around the *S. aureus* abscess. Importantly, pharmacological inhibition or genetic knockout of SLC7A11 promoted the bactericidal function of macrophages, reduced bacterial burden in the bone and improved bone structure in mice with *S. aureus* osteomyelitis. Mechanistically, aberrantly expressed SLC7A11 down-regulated the level of intracellular ROS and reduced lipid peroxidation, contributing to the impaired bactericidal function of macrophages. Interestingly, blocking SLC7A11 further activated expression of PD-L1 via the ROS-NF-κB axis, and a combination therapy of targeting both SLC7A11 and PD-L1 significantly enhanced the efficacy of clearing* S. aureus in vitro* and *in vivo*. Our findings suggest that targeting both SLC7A11 and PD-L1 is a promising therapeutic approach to reprogram the bactericidal function of macrophages and promote bacterial clearance in *S. aureus* osteomyelitis.

## Introduction

Implant-associated infectious osteomyelitis is a severe local inflammation of the bone caused by invasion of pathogenic bacteria 1. Characterized by chronic and persistent inflammation and progressive bone destruction 2, it commonly occurs as a secondary complication following open or closed fractures and surgical internal fixation. Currently, treatment of infectious osteomyelitis relies primarily on early administration of sensitive antibiotics, thorough surgical debridement, and local reconstruction of defects 3, 4. However, these approaches have likely led to unsatisfactory efficacy. Infections often persist, recur, or easily progress into delayed fracture healing, nonunion, and osteonecrosis 5. Severe cases may result even in limb shortening, imposing a substantial burden on the individual victims and the society as a whole 3. Hence, there is an urgent need to investigate the regulatory mechanisms underlying progression of infectious osteomyelitis.

*Staphylococcus aureus* (*S. aureus*), the gram-positive bacterium, is currently the primary pathogen in infectious osteomyelitis 5, 6. During the prolonged host-pathogen interaction process, *S. aureus* has evolved multiple mechanisms to evade immune system surveillance and elimination, including biofilm formation on the surface of implanted materials, secretion of abundant virulence factors, modification of cell wall components to enhance antibiotic resistance, and internalization into host cells or the osteocyte-lacuno canalicular network (OLCN) within bone tissue 7-10. Innate immune cells, mainly including neutrophils, monocytes, and macrophages, possess pattern recognition receptors on their cell surface to recognize pathogen-associated molecular patterns (PAMPs) on the surface of pathogens 11. This recognition process facilitates phagocytosis of pathogens and initiation of immune responses 11, 12. Macrophages serve as the primary reservoir for the long-term intracellular colonization of *S. aureus* 13 and loss of their antimicrobial activity is closely associated with the pathogenesis of *S. aureus*-induced osteomyelitis 14, 15. Our recent research has found that *S. aureus* infection may trigger the activity of PD-1/PD-L1 signaling in macrophages, leading to increased mitophagy and impaired antimicrobial activity 16. Despite the fact that studies on immune checkpoint-targeted therapy in various diseases such as tumors and infections are extensive, preclinical or clinical applications of the therapy remain limited due to complex immune microenvironment and low sensitivity of immunotherapy 17-20. Therefore, further investigation is needed to explore the strategies which may effectively enhance the sensitivity of immune checkpoint blockade therapy in *S. aureus*-induced osteomyelitis.

SLC7A11 is a type of cystine-glutamate transport protein that primarily mediates the reverse transport of extracellular cystine into the cytoplasm 21. Ingested cystine is reduced to cysteine by consuming nicotinamide adenine dinucleotide phosphate-oxidase (NAPDH), making it available for cellular utilization 21, 22. Cysteine acts not only as a fundamental substrate for protein synthesis but also as a rate-limiting precursor for glutathione biosynthesis, which is critical for the maintenance of cellular redox homeostasis 23. Recent studies have indicated that activation of SLC7A11 signaling can induce transition of macrophage into an M2 immunosuppressive phenotype 24, 25. SLC7A11 blockade is advantageous for the host to defend against pathogens, as in malaria and *Mycobacterium tuberculosis*, partly due to its regulation of oxidative-reductive metabolism, such as ROS generation and lipid peroxidation 26, 27. However, the role and mechanism of SLC7A11 in *S. aureus*-induced osteomyelitis remain poorly understood.

This study elucidates a close association between aberrantly activated SLC7A11 signaling and immunosuppressive state of macrophages in *S. aureus*-induced osteomyelitis. Treatment with erastin, a pharmacological inhibitor of SLC7A11, or macrophage-specific knockout of SLC7A11, drove ROS production and lipid peroxidation *in vitro* and *in vivo*, thereby effectively reducing bacterial load and improving bone destruction. It is worth noting that a combination blockade of both SLC7A11 and PD-L1 signaling strongly promoted clearance of* S. aureus* and improved resolution of osteomyelitis in mice. Together, these findings identify SLC7A11 as a novel molecular target in a combination immunotherapy for *S. aureus*-induced osteomyelitis.

## Results

### Pathogenesis of *S. aureus*-induced osteomyelitis is accompanied by an immunosuppressive state in macrophages

Our previous work revealed characteristic histological changes during the progression from acute to subacute to chronic stage of *S. aureus* osteomyelitis in a mouse model of implant-associated osteomyelitis 28. Consistently, here we observed progressive bone destruction during subacute *S. aureus* osteomyelitis in mice. As revealed by H&E staining, small clusters of neutrophils occurred in the marrow space around implantation site at day 3 after infection (Figure S1A and S1C). By day 7, large amounts of neutrophils accumulated around the implantation site, accompanied by the emergence of abscess and fibrosis in the bone marrow cavity, and the metaphyseal trabecular bone became thinner (Figure S1A and S1C). As the infection continued, multiple foci of abscess with progressive fibrosis occupied bone marrow, even spreading to the metaphyseal area of distal femur at day 14. Importantly, metaphyseal trabecular bones were almost completely lost, multiple sequestra appeared in the bone marrow and periosteal reaction progressed in the infected femur at day 14 (Figure S1A and S1C). Quantitative analysis using modified Smeltzer's scoring system revealed progressive histopathological changes in mice femurs with* S. aureus* osteomyelitis (Figure S1B).

As the first line of defense against invading pathogens, macrophages also serve as the primary reservoir for persistent *S. aureus* infection in the host 13, 29. We therefore analyzed the recruitment of macrophages and bacterial burden in the pathogenesis of subacute osteomyelitis in *Lyz2Cre-Tomato* mice.

**Figure 1:**
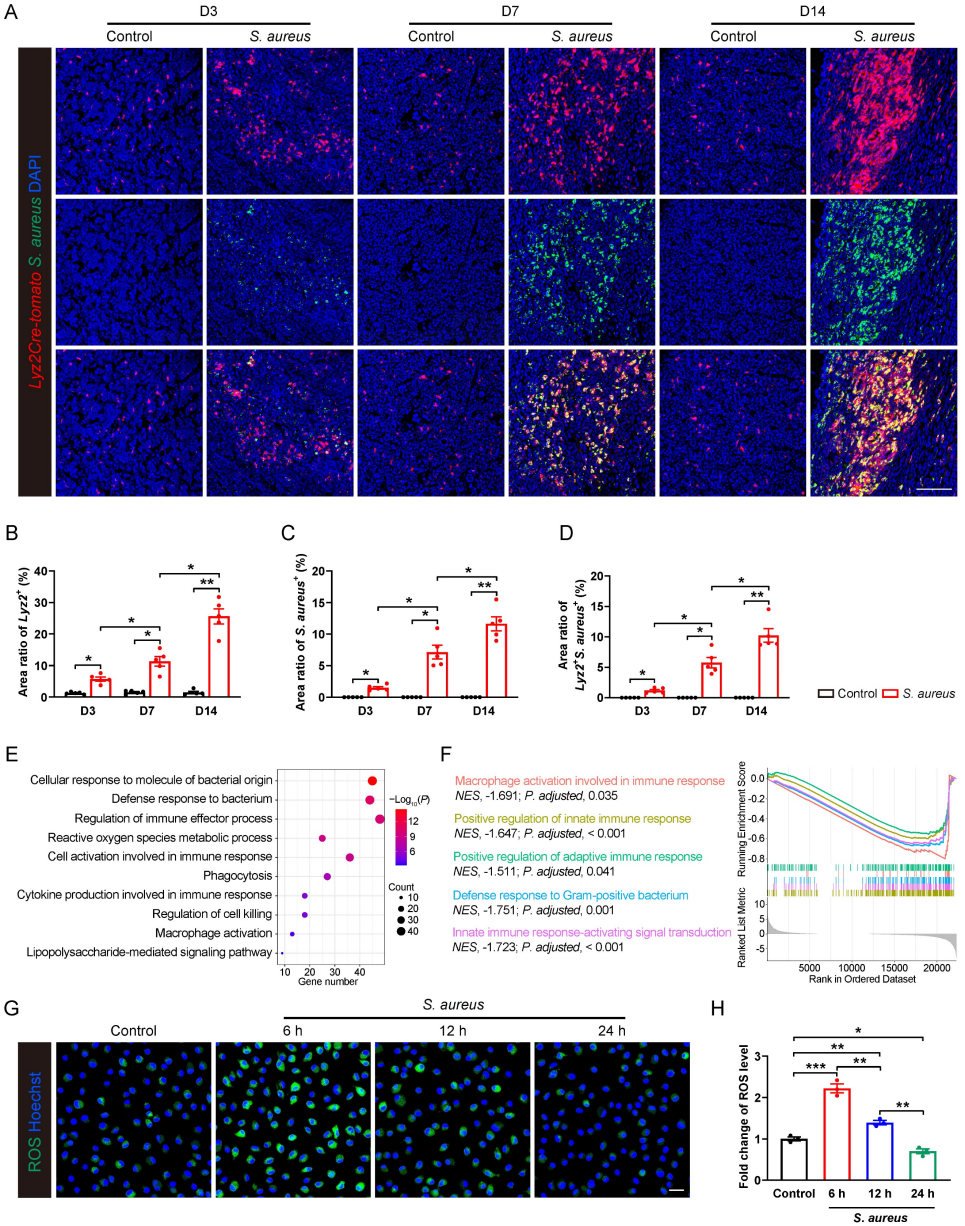
** Pathogenesis of *S. aureus*-induced osteomyelitis is accompanied by an immunosuppressive state in macrophages. (A-D)** Representative immunofluorescence images of *S. aureus* (green) and *Lyz2^+^* macrophages (red) in the femurs from *S. aureus* osteomyelitis mice and control ones. Quantification of the percentages of *Lyz2^+^*, *S. aureus*^+^, and *Lyz2^+^S. aureus*^+^ areas out of total bone marrow area are shown in (B), (C), and (D), respectively. D3, D7, and D14 represent time points of sample collection by days 3, 7, and 14 after surgery, respectively. Scale bar, 100 µm. n = 5/group. **(E)** GO analysis of differentially expressed genes (DEGs) in the transcriptomes of CD11b^+^F4/80^+^ cells from the infected femurs after 14 days of *S. aureus* infection versus that from control ones. GO items with an adjusted *P-value* < 0.05 were considered significantly enriched. **(F)** Significantly down-regulated biological processes of DEGs of transcriptomes in CD11b^+^F4/80^+^ cells from the infected femurs compared with that from the control femurs. GSEA items with a normalized enrichment score ≥ 1, a *P-value* < 0.05, and a Benjamini-Hochberg false discovery rate < 0.25 were considered significantly enriched. **(G and H)** Representative images and quantitative results of ROS levels in BMDMs after *S. aureus* infection at different time points (0, 6, 12 and 24 hours). Scale bar, 20 µm. n = 3/group. Data are shown as means ± SEM. One-way ANOVA with Fisher's LSD *post hoc* test (H) or Dunnett's T3 *post hoc* test (B, C, D) was used. *P < 0.05, **P < 0.01, ***P < 0.001.

We found a significant recruitment of* Lyz2^+^* macrophages around abscess lesion as early as day 3 post-infection, and their numbers progressively increasing at days 7 and 14 (Figure 1A and 1B), indicating an active innate immune response of the host. However, *S. aureus* expanded considerably in the bone marrow as the osteomyelitis progressed (Figure 1A and 1C). Moreover, a substantial increase in the co-localization of *S. aureus* with *Lyz2^+^* macrophages was observed at day 7, further accentuating at day 14 (Figure 1A and 1D), indicating a compromised antimicrobial function of *Lyz2^+^* macrophages during the subacute stage of *S. aureus* osteomyelitis in mice.

To characterize the gene expression profiles associated with the impaired antimicrobial function of macrophages, we performed RNA sequencing on CD11b^+^F4/80^+^ macrophages sorted from control and *S. aureus*-infected femurs of mice by 14 days post-infection. Gene ontology (GO) analysis revealed that the differentially expressed genes (DEGs) were mainly involved in immune regulation and bacterial defense response (Figure 1E). Further gene set enrichment analysis (GSEA) indicated a significant inhibition of multiple biological processes, including macrophage activation, innate immune response, adaptive immune response, and Gram-positive bacterial defense response (Figure 1F). Given that ROS generation is critical to effective antimicrobial activity of activated macrophages 30, 31, we investigated the dynamic ROS changes in primary murine bone marrow-derived macrophages (BMDMs) following *S. aureus* infection. Results showed that the level of ROS was significantly increased at 6 hours post-infection, followed by a marked decrease at 12 and 24 hours (Figure 1G and 1H). Taken together, the above findings indicate that the antimicrobial activity of macrophages may be impaired during the subacute period of *S. aureus* osteomyelitis when *S. aureus* evades macrophage killing and establishes intracellular residency, leading to chronic persistent inflammation and recurrent infections.

### SLC7A11 expression is up-regulated in macrophages after *S. aureus* infection

**Figure 2:**
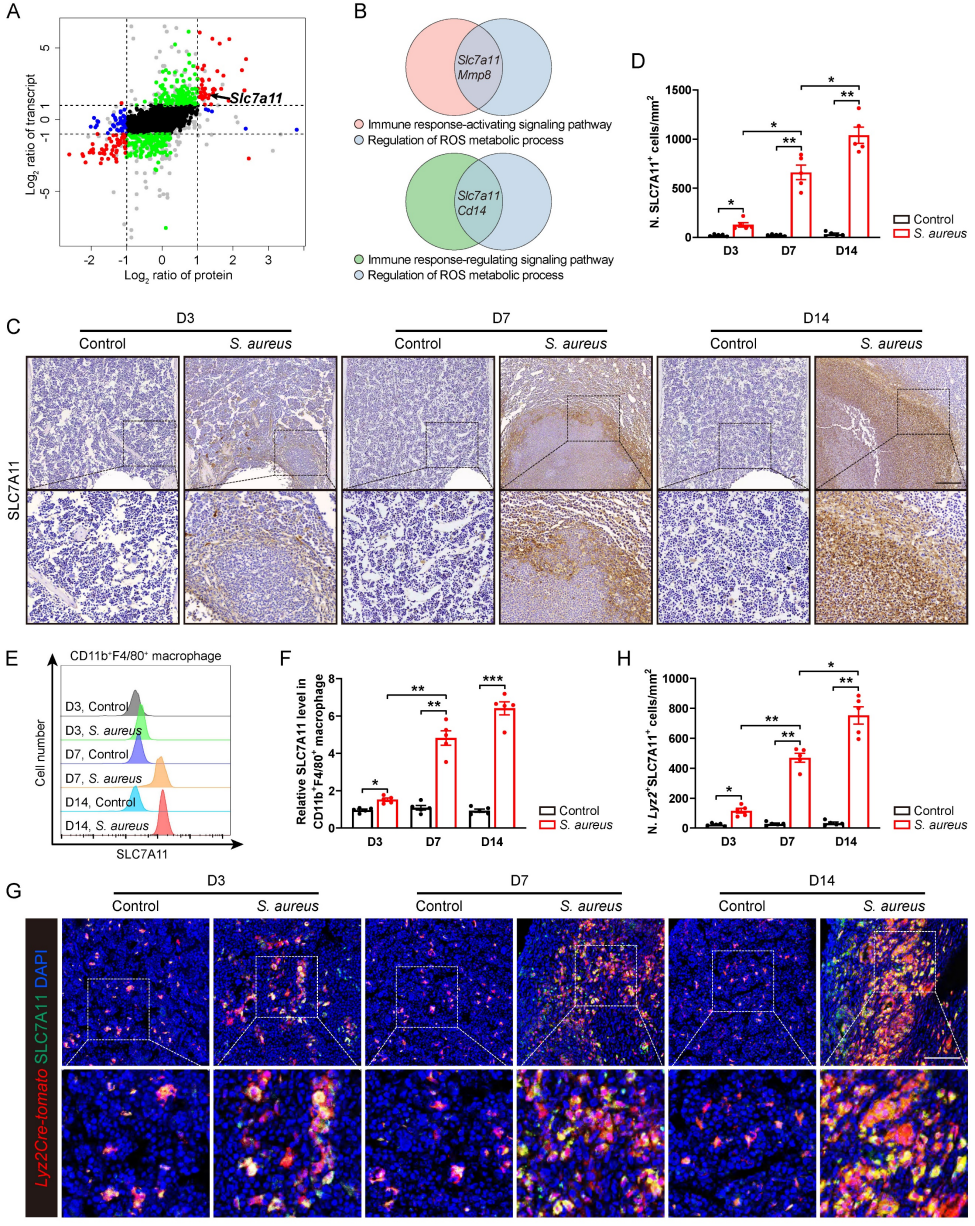
** SLC7A11 expression is up-regulated in macrophages after *S. aureus* infection. (A)** Nine-quadrant plot of DEGs in the transcriptomes and translatomes of CD11b^+^F4/80^+^ cells from the femurs of *S. aureus* osteomyelitis mice and control ones by day 14 after surgery. **(B)** Venn diagram of DEGs associated with immune response signaling pathway and ROS metabolic process. **(C and D)** Representative immunohistochemical images of SLC7A11 in the femurs of *S. aureus* osteomyelitis mice and control ones. Quantification of the number of SLC7A11^+^ cells per mm^2^ tissue area (N. SLC7A11^+^ cells) is shown in (D). D3, D7, and D14 represent time points of sample collection by days 3, 7, and 14 after surgery, respectively. Scale bar, 250 µm. n = 5/group.** (E and F)** Representative images of flow cytometry and quantification of SLC7A11 levels in CD11b^+^F4/80^+^ macrophages from *S. aureus* osteomyelitis mice and control ones. D3, D7, and D14 represent time points of sample collection by days 3, 7, and 14 after surgery, respectively. n = 5/group. **(G and H)** Representative immunofluorescent images of SLC7A11 (green) and *Lyz2Cre-Tomato* (red) in the femurs from *S. aureus* osteomyelitis mice and control ones. Quantification of the numbers of *Lyz2*- and SLC7A11-double positive cells per mm^2^ tissue area (N. *Lyz2*^+^SLC7A11^+^ cells) is shown in (H). Scale bar, 100 µm. n = 5/group. Data are shown as means ± SEM. One-way ANOVA with Dunnett's T3 *post hoc* test was used. *P < 0.05, **P < 0.01, ***P < 0.001.

In order to elucidate the underlying molecular mechanisms whereby the antimicrobial function of macrophages is impaired in *S. aureus*-induced osteomyelitis, we performed proteomic mass spectrometry analysis on flow-sorted CD11b^+^F4/80^+^ macrophages from both control and infected femurs on postoperative day 14. There were 1,172 and 155 DEGs (foldchange > 2) in transcriptomes and translatomes, respectively (Figure S2A and S2B). We further categorized DEGs at the transcriptomes and translatomes according to their expression levels. 39 DEGs were both transcriptionally and translationally higher in the macrophages from *S. aureus*-infected bones than those in the control ones (Figure 2A). GO analysis revealed that these genes predominantly participate in the immune response-activating signaling pathway, immune response-regulating signaling pathway, and ROS metabolic process (Figure S2C). Notably, SLC7A11 emerged as a key player regulating both macrophage immune response and ROS metabolic processes (Figure 2B).

Next, we focused on SLC7A11 because it participates in maintaining the intracellular redox homeostasis under various physiological and pathological conditions 21, 23 as one of the functional subunits of cystine transporters. To confirm that *S. aureus* infection might up-regulate the expression of SLC7A11 *in vivo*, we detected the levels of SLC7A11 by immunohistochemistry. In mice models of acute and subacute implant-associated osteomyelitis, we observed a slightly increased protein level of SLC7A11 at day 3 post-infection, peaking at days 7 and 14 post-infection. Interestingly, SLC7A11 exhibited a characteristic distribution around the abscess in bone marrow (Figure 2C and 2D). Now that neutrophils, monocytes, and macrophages are important cells in the early phase of defense against *S. aureus* infection in the host's innate immune system 13, we examined the expression patterns of SLC7A11 in these three cell populations within infected mouse bones. Flow cytometry analysis revealed a significant increase in SLC7A11 expression levels in CD11b^+^F4/80^+^ macrophages as early as day 3 post-infection, which further increased as *S. aureus* osteomyelitis progressed (Figure 2E and 2F). However, *S. aureus* infection induced only marginal up-regulation of SLC7A11 expression in CD11b^+^Ly6G^+^ neutrophils by day 14 post-infection, while expression levels remained unchanged in CD11b^+^Ly6C^+^ monocytes throughout the entire infection course (Figure S2D-S2G). Double immunofluorescence staining also showed a significant increase in the number of *Lyz2^+^*SLC7A11^+^ cells around the abscesses in the bone marrow cavity (Figure 2G and 2H). These results consistently indicate that macrophages may be the main cell subset responsible for the robust expression of SLC7A11 in *S. aureus*-induced osteomyelitis.

### Inhibition of SLC7A11 enhances the bactericidal capacity of macrophages by inducing ROS generation and lipid peroxidation

We then investigated whether *S. aureus* infection directly stimulates the expression of SLC7A11 in macrophages *in vitro*. Primary BMDMs were infected with *S. aureus* suspensions at various MOI (0, 0.1, 1 and 10) for one hour. After extracellular bacteria were killed and removed, the BMDMs were incubated for an additional 12 hours.

**Figure 3:**
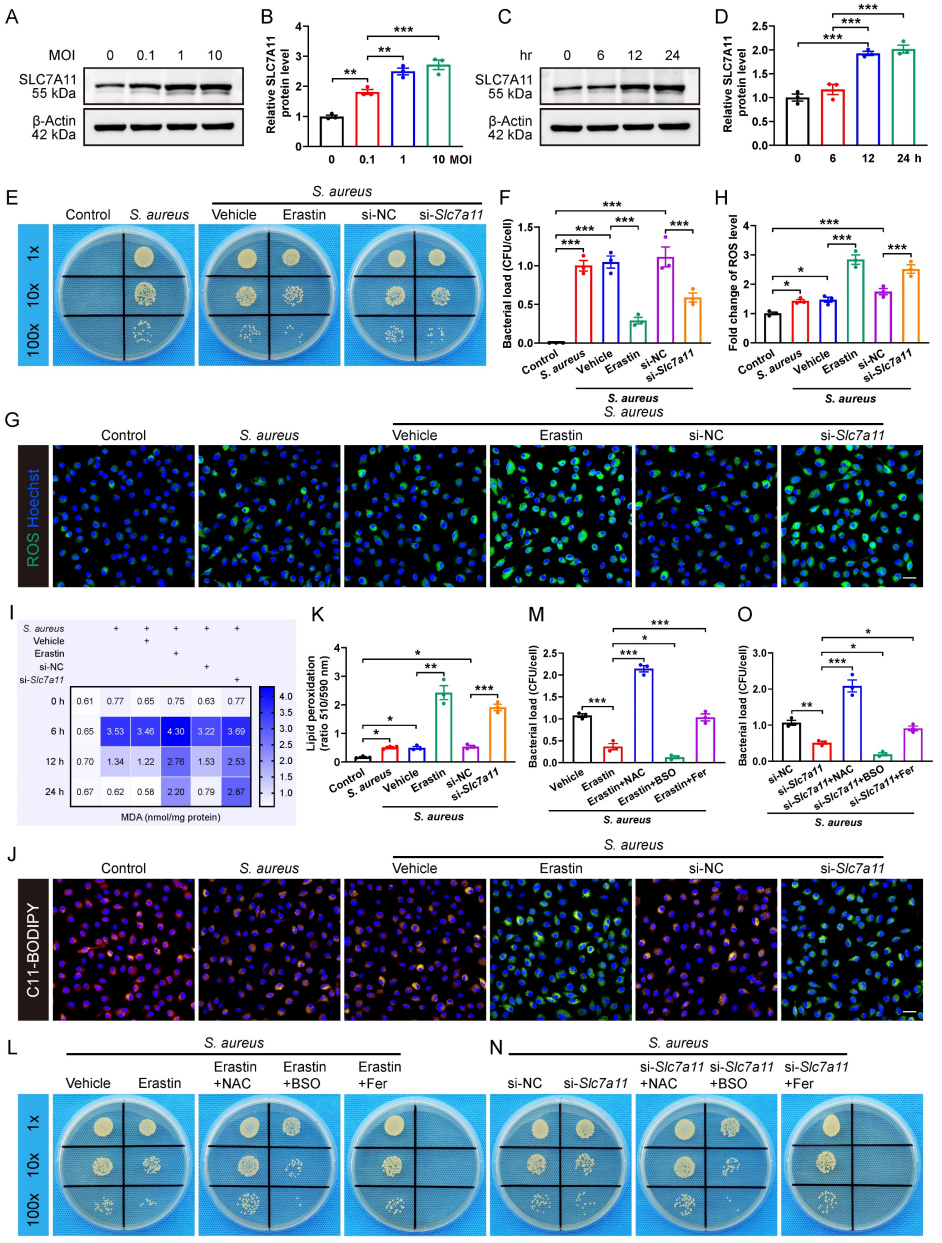
** Inhibition of SLC7A11 enhances the bactericidal capacity of macrophages by inducing ROS generation and lipid peroxidation. (A and B)** Representative images and quantification of western blots for SLC7A11 in BMDMs infected with *S. aureus* at different MOI (0, 0.1, 1, and 10) for 12 hours. **(C and D)** Representative images and quantification of western blots for SLC7A11 in BMDMs infected with *S. aureus* (MOI = 10) at different time points (0, 6, 12, and 24 hours). **(E and F)** Representative images and quantification of bacterial burden in BMDMs at 12 hours post-infection with *S. aureus* (MOI = 10) in the presence of erastin (5 µM) or siRNA targeting *Slc7a11* (si-*Slc7a11*, 100 nM). n = 3/group. **(G and H)** Representative images and quantification of ROS levels in BMDMs after 12 hours of *S. aureus* infection in the presence of erastin (5 µM) or si-*Slc7a11* transfection (100 nM). Scale bar, 20 µm. n = 3/group. **(I)** Quantification of malondialdehyde (MDA) levels in BMDMs at indicated times after* S. aureus* infection in the presence of erastin (5 µM) or si-*Slc7a11* transfection (100 nM). Mean values of three independent experiments are shown in the heatmap. n = 3/group. **(J and K)** Representative images and quantification of lipid peroxidation levels in BMDMs at 12 hours after *S. aureus* infection in the presence of erastin (5 µM) or si-*Slc7a11* transfection (100 nM). Reduced state lipids are shown in red fluorescence, and oxidized state lipids are shown in green fluorescence. Scale bar, 20 µm. n = 3/group. **(L and M)** Representative images and quantification of bacterial burden in BMDMs at 12 hours post-infection. Prior to *S. aureus* infection, BMDMs were pre-treated with vehicle (DMSO) alone, or erastin (5 µM) alone, or a combination of erastin (5 µM) and ROS scavengers (NAC, 500 µM), or a combination of erastin (5 µM) and ROS inducers (BSO, 100 µM), or a combination of erastin (5 µM) and lipid peroxidation inhibitors (Fer, ferrostatin-1, 10 µM) for one hour. n = 3/group. **(N and O)** Representative images and quantification of bacterial burden in BMDMs at 12 hours post-infection. After being transfected with si-NC or si-*Slc7a11* for 48 hours, BMDMs were pre-treated with NAC, BSO or Fer for another one hour, followed by infection with *S. aureus* at a MOI of 10. n = 3/group. Data are shown as means ± SEM. One-way ANOVA with Fisher's LSD *post hoc* test (B, D, F, H, M, O) or Dunnett's T3 *post hoc* test (K) was used. *P < 0.05, **P < 0.01, ***P < 0.001.

The results revealed that *S. aureus* infection significantly up-regulated the expression of SLC7A11 in macrophages in a concentration-dependent manner (Figure 3A and 3B). Next, we evaluated the time-dependent effect of *S. aureus* (MOI = 10) on the protein level of SLC7A11 in BMDMs. Interestingly, the protein level of SLC7A11 in BMDMs was not altered at 6 hours post-infection. However, significant increases in the protein level of SLC7A11 were observed at 12 and 24 hours post-stimulation (Figure 3C and 3D). The time-dependent changes in the expression level of SLC7A11 were exactly opposite to the trend of intracellular ROS levels in macrophages observed in our previous work 16, indicating a potential link between the aberrant expression of SLC7A11 and the impaired bactericidal capacity of macrophages. To corroborate the regulation of antimicrobial activity in macrophages by SLC7A11, BMDMs were treated with erastin, a pharmacological inhibitor of SLC7A11, or with siRNA-mediated *Slc7a11* silencing (si-*Slc7a11*) before infection with *S. aureus*. As shown in Figure S3A and S3B, treatment with erastin or si-*Slc7a11* effectively blocked the mRNA expression of SLC7A11 induced by *S. aureus* infection. As expected, we observed a considerably decreased bacterial burden in macrophages by erastin or si*-Slc7a11* treatment (Figure 3E and 3F).

It is known that the antibacterial activity of macrophages is closely related to the ROS generation 30, 31. To evaluate the role of SLC7A11 in regulating intracellular ROS levels in response to *S. aureus* infection, we used fluorescence probe DCFH-DA for labeling ROS. There was only a minor increase in the levels of ROS in BMDMs after 12 hours of *S. aureus* infection (Figure 3G and 3H), which is consistent with our previous finding that ROS exhausted in macrophages following persistent *S. aureus* infection* in vitro* 16. Intriguingly, blocking SLC7A11 by erastin treatment or si*-Slc7a11* significantly rescued the levels of intracellular ROS suppressed after 12 hours of *S. aureus* infection (Figure 3G and 3H). Recent studies have shown that intracellular ROS-induced lipid peroxidation of polyunsaturated fatty acids in the host helped eliminate various pathogens, including *S. aureus* 26, 30, 32. Additionally, the changes in content of malondialdehyde (MDA), a stable end product of lipid peroxidation, directly reflect the degrees of lipid peroxidation 33. As expected, we found that the intracellular levels of MDA were significantly decreased in macrophages after 12 hours of infection but the effect could be blocked by erastin treatment or si-*Slc7a11* (Figure 3I). To confirm the role of SLC7A11 in regulation of the levels of lipid peroxidation, we used a C11-BODIPY 581/591 fluorescence probe to label the intracellular lipid peroxidation. After 12 hours of* S. aureus* infection, the red fluorescence shifted to yellow, indicating a slightly increased level of lipid peroxidation in BMDMs. Furthermore, blocking SLC7A11 by erastin treatment or si-*Slc7a11* significantly increased the level of lipid peroxidation, evidenced by a significant green emission from the oxidized C11-BODIPY (Figure 3J and 3K). Given the importance of proinflammatory cytokines, such as TNF-ɑ, IL-1β, and IL-6 in protective immunity against* S. aureus* infection 34, we investigated the role of SLC7A11 in regulating inflammatory responses. We found that *S. aureus* infection significantly up-regulated the expression of TNF-ɑ, IL-1β, and IL-6 mRNA, and blocking SLC7A11 further enhanced the expression of TNF-ɑ and IL-6 mRNA (Figure S3C-S3E).

We next examined whether SLC7A11 might suppress the antimicrobial activity of macrophages by down-regulating the levels of ROS and lipid peroxidation. The results demonstrated that buthionine sulfoximine (BSO), a ROS inducer, synergistically enhanced the bactericidal capability of macrophages in conjunction with erastin treatment or si*-Slc7a11*. Conversely, both ROS scavengers (N-acetylcysteine, NAC) and lipid peroxidation inhibitors (ferrostatin-1) exerted antagonistic effects on antimicrobial activity of BMDMs, leading to a significant increase in bacterial load in the cells (Figure 3L-3O). Together, these findings suggest that aberrant expression of SLC7A11 may destroy the antimicrobial activity of macrophages by suppressing ROS generation and lipid peroxidation.

### Blocking SLC7A11 ameliorates the pathogenesis of *S. aureus* osteomyelitis in mice

**Figure 4:**
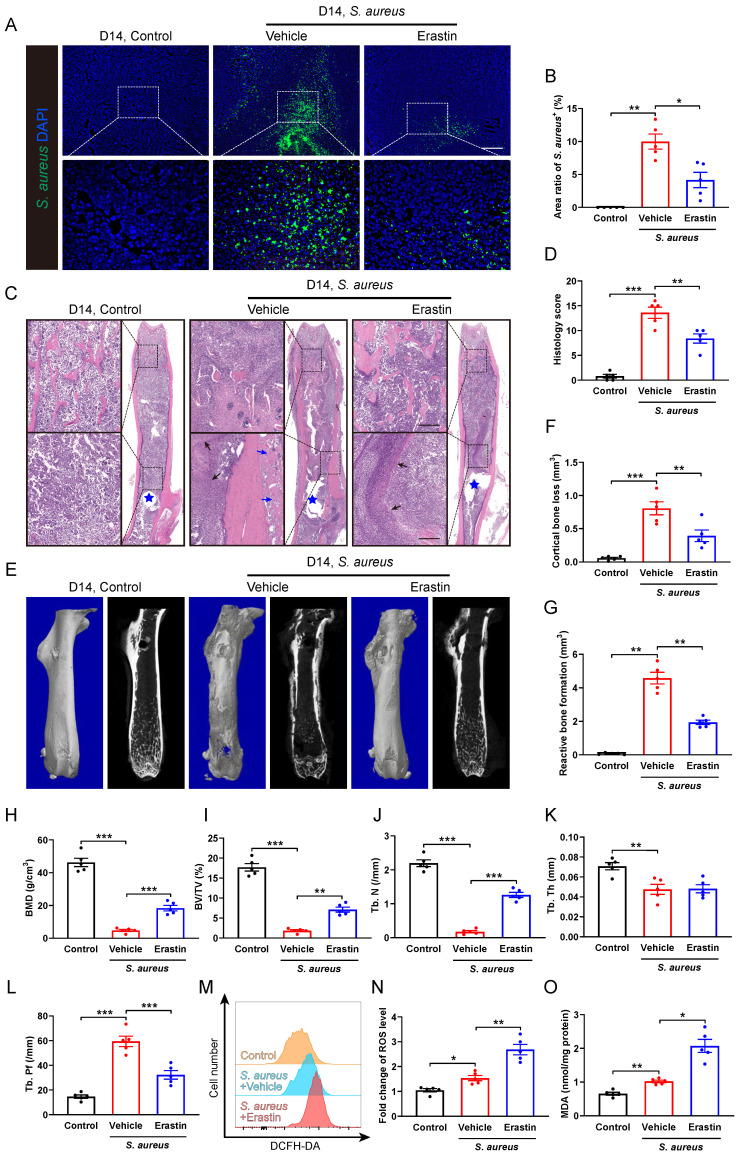
** Erastin treatment ameliorates the pathogenesis of *S. aureus* osteomyelitis in mice. (A and B)** Representative immunofluorescence images of *S. aureus* in the femurs from *S. aureus* osteomyelitis mice treated with vehicle or erastin (30 mg/kg of body weight, once every two days) and control ones. Quantification of the percentage of *S. aureus*^+^ areas out of total bone marrow area is shown in (B). Mice were euthanized and the right femurs were collected by day 14 after implant-associated *S. aureus* osteomyelitis surgery. Scale bar, 200 µm. n = 5/group. **(C and D)** Representative images and histological score of H&E staining of the femurs from *S. aureus* osteomyelitis mice treated with vehicle or erastin and control ones by day 14 after surgery. Blue stars show the position of the implant in bone marrow cavity. Dark arrows show abscess in bone marrow cavity, and blue arrows show reactive new bone formation. Scale bar, 200 µm. n = 5/group. **(E)** Representative images of three-dimensional and coronal micro-CT of the femurs from *S. aureus* osteomyelitis mice treated with vehicle or erastin and control ones by day 14 after surgery. **(F and G)** Quantitative analysis of cortical bone loss and reactive bone formation of the femurs from *S. aureus* osteomyelitis mice treated with vehicle or erastin and control ones by day 14 after surgery. n = 5/group. **(H-L)** Quantitative analysis of trabecular bone mineral density (BMD), bone fraction (BV/TV), trabecular number (Tb. N), trabecular thickness (Tb. Th) and trabecular bone pattern factor (Tb. Pf) of the femurs from *S. aureus* osteomyelitis mice treated with vehicle or erastin and control ones by day 14 after surgery. n = 5/group. **(M and N)** Representative images of flow cytometry and quantification of ROS levels in CD11b^+^F4/80^+^ macrophages from *S. aureus* osteomyelitis mice treated with vehicle or erastin and control ones by day 14 after surgery. n = 5/group. **(O)** Quantification of malondialdehyde (MDA) levels in CD11b^+^F4/80^+^ macrophages from *S. aureus* osteomyelitis mice treated with vehicle or erastin and control ones by day 14 after surgery. n = 5/group. Data are shown as means ± SEM. One-way ANOVA with Fisher's LSD *post hoc* test (D, F, H, J, K, L) or Dunnett's T3 *post hoc* test (B, G, I, N, O) was used. *P < 0.05, **P < 0.01, ***P < 0.001.

We subsequently investigated the role of SLC7A11 in the pathological development of *S. aureus* osteomyelitis *in vivo*. A model of implant-associated osteomyelitis was established in the mice, which were randomly divided into three groups subjected to either vehicle or erastin treatment. Erastin treatment dramatically suppressed the protein levels of SLC7A11 in the femurs of *S. aureus*-infected mice compared with vehicle treatment (Figure S4). Immunofluorescence staining revealed a dramatic reduction in the* S. aureus^+^* area in the infected femur of mice treated with erastin compared with vehicle-treated ones (Figure 4A and 4B), demonstrating a potential effect of blocking SLC7A11 on reducing bacterial burden in bone. Histopathological examination revealed extensive abscess formation in the entire bone marrow cavity, and disorganized and diminished trabecular structures in the *S. aureus*-infected mice treated with vehicle, while a significant improvement in bone structure was observed in the mice receiving erastin treatment (Figure 4C and 4D). Consistent with these findings, micro-CT imaging showed that *S. aureus* infection led to substantial cortical bone defects and cortical bone loss around the femoral nail canal, accompanied by reactive cortical bone formation, while treatment with erastin significantly ameliorated bone destruction and reduced reactive cortical bone formation (Figure 4E-4G).

Analysis of the trabecular bone microstructure showed significant increases in bone mineral density (BMD), ratio of bone volume to total volume (BV/TV) and trabecular number (Tb. N), and a reduction in trabecular bone pattern factor (Tb. Pf) in the erastin-treated mice (Figure 4E, 4H-4L). Furthermore, flow cytometry analysis showed that inhibition of SLC7A11 by erastin markedly elevated the levels of ROS and lipid peroxidation in CD11b^+^F4/80^+^ macrophages within the bone marrow cavity after 14 days of* S. aureus* infection (Figure 4M-4O). These results suggest that pharmacological inhibition of SLC7A11 may enhance bactericidal activity of macrophages by elevating ROS levels, thereby ameliorating the pathogenesis of *S. aureus* osteomyelitis in mice.

**Figure 5:**
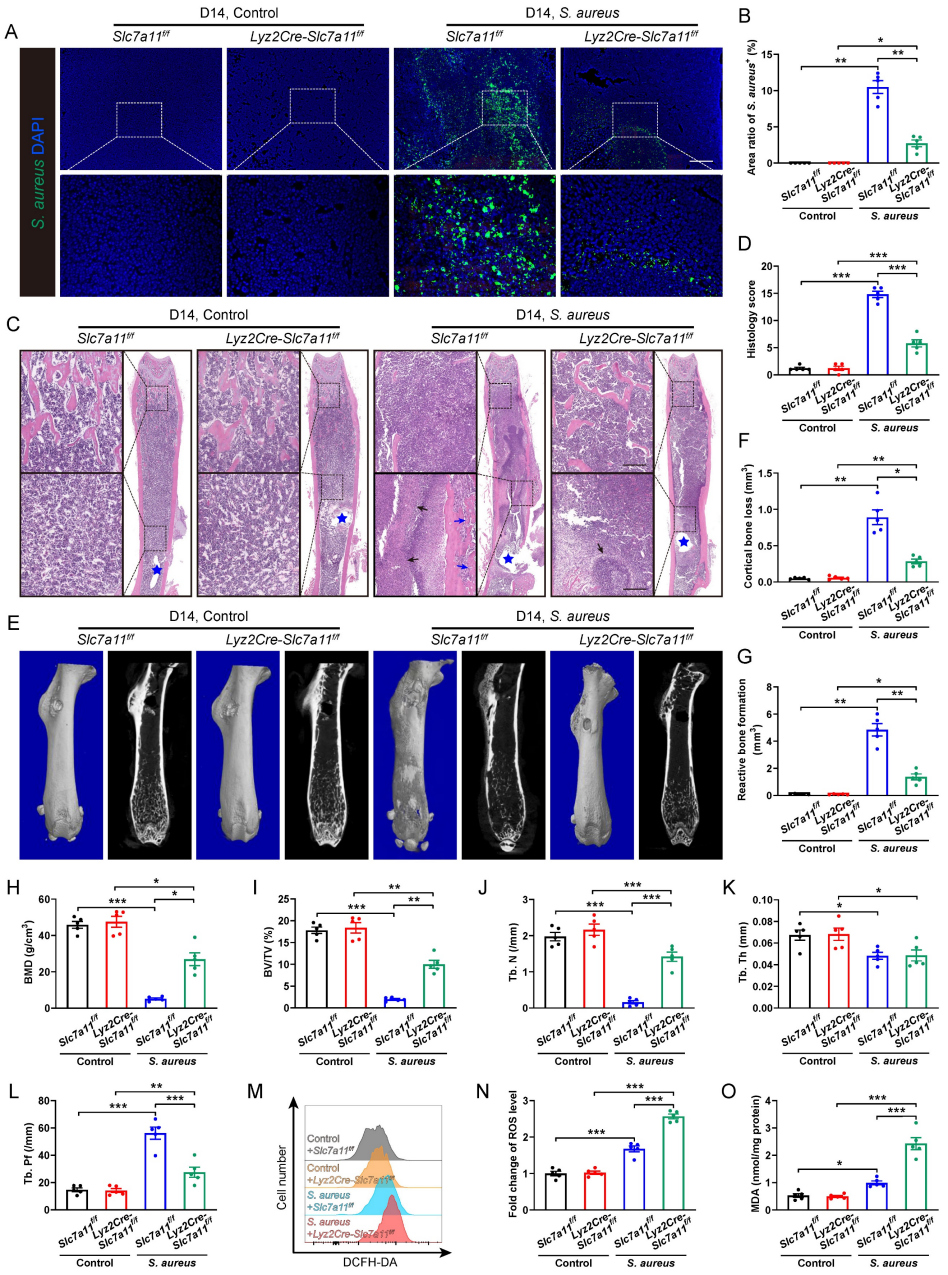
** Macrophage-specific knockout of *Slc7a11* ameliorates the pathogenesis of *S. aureus* osteomyelitis in mice. (A and B)** Representative immunofluorescence images of *S. aureus* in the femurs from *Lyz2Cre-Slc7a11^f/f^* mice and *Slc7a11^f/f^* mice. Quantification of the percentage of *S. aureus*^+^ areas out of total bone marrow area is shown in (B). Mice were euthanized and the right femurs were collected by day 14 after implant-associated *S. aureus* osteomyelitis surgery. Scale bar, 200 µm. n = 5/group. **(C and D)** Representative images and histological score of H&E staining of the femurs from *Lyz2Cre-Slc7a11^f/f^* mice and *Slc7a11^f/f^* mice by day 14 after surgery. Blue stars show the position of the implant in bone marrow cavity. Dark arrows show abscess in bone marrow cavity, and blue arrows show reactive new bone formation. Scale bar, 200 µm. n = 5/group. **(E)** Representative images of three-dimensional and coronal micro-CT of the femurs from *Lyz2Cre-Slc7a11^f/f^* mice and *Slc7a11^f/f^* mice by day 14 after surgery. **(F and G)** Quantitative analysis of cortical bone loss and reactive bone formation of the femurs from *Lyz2Cre-Slc7a11^f/f^* mice and *Slc7a11^f/f^* mice by day 14 after surgery. n = 5/group. **(H-L)** Quantitative analysis of trabecular bone mineral density (BMD), bone fraction (BV/TV), trabecular number (Tb. N), trabecular thickness (Tb. Th) and trabecular bone pattern factor (Tb. Pf) of the femurs from *Lyz2Cre-Slc7a11^f/f^* mice and *Slc7a11^f/f^* mice by day 14 after surgery. n = 5/group. **(M and N)** Representative images of flow cytometry and quantification of ROS levels in CD11b^+^F4/80^+^ macrophages from *Lyz2Cre-Slc7a11^f/f^* mice and *Slc7a11^f/f^* mice by day 14 after surgery. n = 5/group. **(O)** Quantification of malondialdehyde (MDA) levels in CD11b^+^F4/80^+^ macrophages from *Lyz2Cre-Slc7a11^f/f^* mice and *Slc7a11^f/f^* mice by day 14 after surgery. n = 5/group. Data are shown as means ± SEM. Two-way ANOVA with Fisher's LSD *post hoc* test (J, K, L, N, O) or Dunnett's T3 *post hoc* test (B, D, F, G, H, I) was used. *P < 0.05, **P < 0.01, ***P < 0.001.

To confirm the role of SLC7A11 in macrophages after persistent *S. aureus* infection, *lysozyme2 (Lyz2)-Cre* (*Lyz2Cre*) mice were mated with *Slc7a11^flox/flox^* (*Slc7a11^f/f^*) mice to generate* Lyz2Cre-Slc7a11^f/f^* mice, in which* Slc7a11* was specifically knocked out in the macrophages. The breeding strategy and gene identification are depicted in Figure S5A and S5B. Before comparing the severity of *S. aureus* osteomyelitis in *Lyz2Cre-Slc7a11^f/f^* mice with that in *Slc7a11^f/f^* mice, we evaluated the protein levels of SLC7A11 in BMDMs from these mice. Under non-infected condition, the protein levels of SLC7A11 in BMDMs derived from *Lyz2Cre-Slc7a11^f/f^* mice were significantly reduced compared with those from *Slc7a11^f/f^* mice. Moreover, *S. aureus* infection could not activate the expression of SLC7A11 in BMDMs from *Lyz2Cre-Slc7a11^f/f^* mice as it did in BMDMs from *Slc7a11^f/f^* mice (Figure S5C and S5D). Immunohistochemical staining confirmed the strikingly down-regulated expression of SLC7A11 in the area around abscess of infected femurs from *Lyz2Cre-Slc7a11^f/f^* mice (Figure S5E and S5F). Compared with* Slc7a11^f/f^* mice, *Lyz2Cre-Slc7a11^f/f^* mice displayed a notable decrease in bacterial burden, which presented as reduced* S. aureus^+^* area within the bone marrow cavity (Figure 5A and 5B). Histological staining and histopathological scoring confirmed the positive effect of macrophage-specific knocking out of *Slc7a11* on the bone structure of mice with *S. aureus* osteomyelitis (Figure 5C and 5D). Specifically, under *S. aureus* osteomyelitis condition, *Slc7a11^f/f^* mice showed deformed femurs with extensive abscess formation in the whole bone cavity, reactive new bone formation around cortical bone, and loss of trabecular bone in epiphyseal area, while the infected femurs in the *Lyz2Cre-Slc7a11^f/f^* mice showed a relatively normal shape, limited abscess formation around the implant and improved trabecular bone structure (Figure 5C). Consistently, micro-CT data demonstrated a noticeable decrease in cortical bone loss and reactive bone formation around the nail in the infected femurs of *Lyz2Cre-Slc7a11^f/f^* mice (Figure 5E-5G). Moreover, the trabecular bone microstructure of the distal femur was substantially improved in the infected femurs of *Lyz2Cre-Slc7a11^f/f^* mice, as revealed by a striking increase in BMD and BV/TV with significantly improved Tb. N and Tb. Pf (Figure 5H-5L). Consistent with the effect of the pharmacological inhibitor erastin on ROS levels in *S. aureus*-infected osteomyelitis mice, specific knockout of *Slc7a11* in macrophages significantly enhanced ROS production and lipid peroxidation metabolism in mice with *S. aureus* osteomyelitis (Figure 5M-5O). Taken together, these results suggest that aberrant expression of SLC7A11 may have detrimental effects on bacterial clearance by suppressing ROS levels in macrophages in *S. aureus*-induced osteomyelitis mice.

### Blocking SLC7A11 promotes PD-L1 expression via the ROS-NF-κB axis in macrophages after *S. aureus* infection

**Figure 6:**
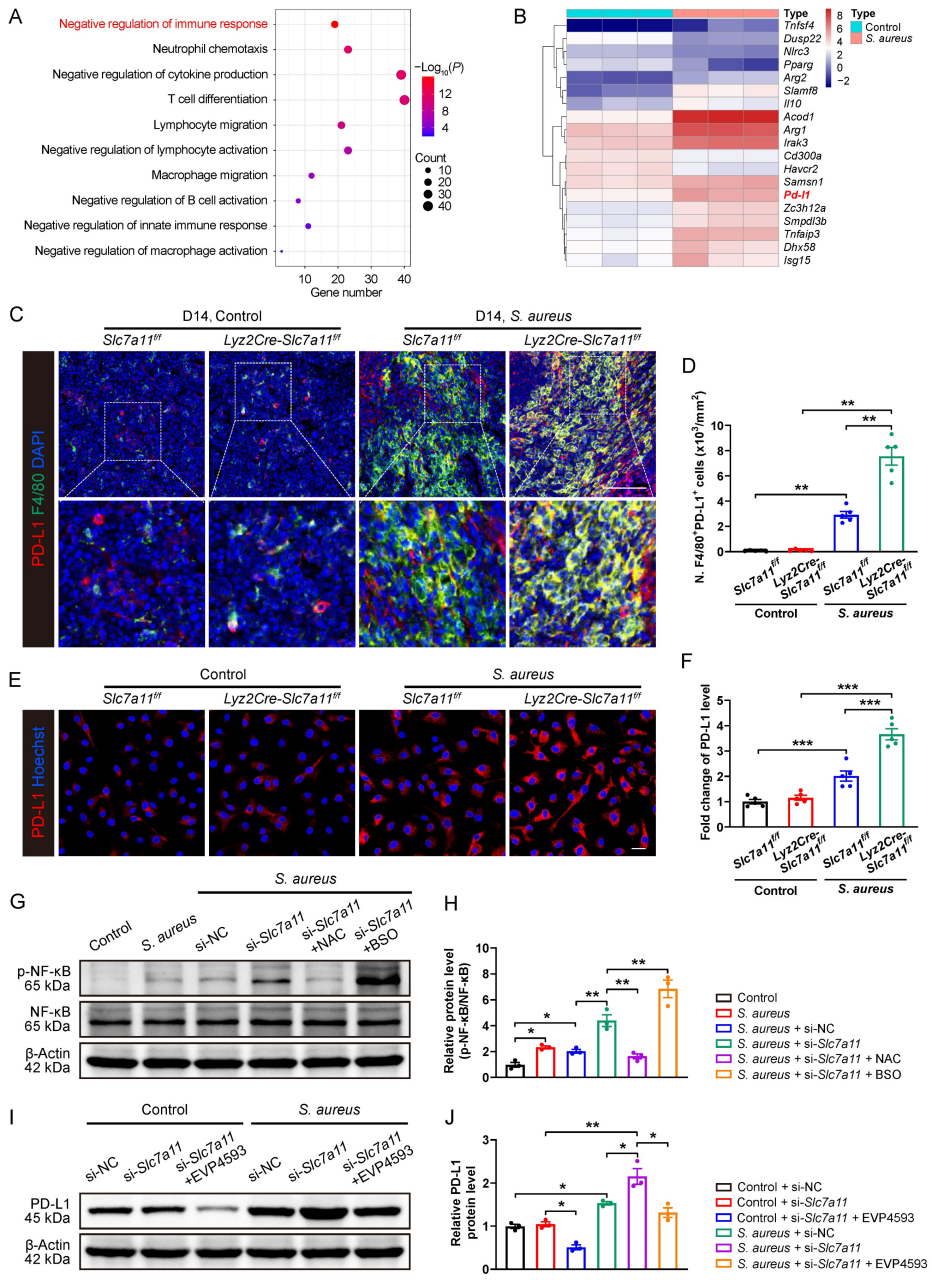
** Blocking SLC7A11 promotes PD-L1 expression via the ROS-NF-κB axis in macrophages after *S. aureus* infection. (A)** GO analysis of DEGs in transcriptomes of CD11b^+^F4/80^+^ cells from femurs of *S. aureus-*infected mice and control ones. GO items with an adjusted *P-value* < 0.05 were considered significantly enriched. **(B)** Heatmap of the DEGs in the GO item “negative regulation of immune response”. **(C and D)** Representative immunofluorescent images of F4/80 (green) and PD-L1 (red) in the femurs from *Lyz2Cre-Slc7a11^f/f^* mice and *Slc7a11^f/f^* mice by day 14 after surgery. Quantification of the numbers of F4/80- and PD-L1-double positive cells per mm^2^ tissue area (N. F4/80^+^PD-L1^+^ cells) is shown in (D). Scale bar, 100 µm. n = 5/group. **(E and F)** Representative images and quantification of immunofluorescence staining for PD-L1 in BMDMs isolated from the femurs of *Lyz2Cre-Slc7a11^f/f^* mice and *Slc7a11^f/f^* mice. Cells were infected with *S. aureus* at a MOI of 10 for 12 hours. Scale bar, 20 µm. n = 5/group. **(G and H)** Representative images and quantification of western blots for the phosphorylation levels of NF-κB in BMDMs with indicated treatments. After being transfected with si-NC (100 nM) or si-*Slc7a11* (100 nM) for 48 hours, BMDMs were pre-treated with or without ROS scavengers (NAC, 500 µM) and ROS inducers (BSO, 100 µM) for another one hour, followed by infection with *S. aureus* at a MOI of 10 for 12 hours. n = 3/group. **(I and J)** Representative images and quantification of western blots for PD-L1 in BMDMs with indicated treatments. After being transfected with si-NC (100 nM) or si-*Slc7a11* (100 nM) for 48 hours, BMDMs were pre-treated with or without NF-κB inhibitors (EVP4593, 0.1 µM) for another one hour, followed by treatment with *S. aureus* at a MOI of 10 or an equal volume of PBS for 12 hours. n = 3/group. Data are shown as means ± SEM. One-way ANOVA with Fisher's LSD *post hoc* test (H), and two-way ANOVA with Fisher's LSD *post hoc* test (F, J) or Dunnett's T3 *post hoc* test (D) was used. *P < 0.05, **P < 0.01, ***P < 0.001.

The data so far have demonstrated that, similar to PD-1/PD-L1 signaling 16, SLC7A11 suppresses antimicrobial function of macrophages by reducing ROS in response to persistent *S. aureus* infection. Next, we explored whether aberrant expression of SLC7A11 might contribute to overactivation of PD-1/PD-L1 signaling in macrophages in response to* S. aureus* infection. We analyzed the transcriptomic and proteomic data of CD11b^+^F4/80^+^ macrophages from the bone marrow of mice with *S. aureus*-infected osteomyelitis at day 14. GO analysis of DEGs in the transcriptomes revealed that they were mainly associated with negative regulation of immune response, cytokine production, and regulation of immune cell activation (Figure 6A). We subsequently analyzed DEGs related to negative regulation of immune response and identified a significant up-regulation of PD-L1 mRNA in the macrophages from *S. aureus*-infected bones (Figure 6B).

Consistent with the transcription level findings, GO analysis of DEGs in the translatomes data also showed enrichment in the process of negative regulation of cell activation (Figure S6A), among which the protein expression of PD-L1 were significantly up-regulated in macrophages from *S. aureus*-infected bones (Figure S6B). In consistent with our previous finding 16, the mRNA expression of PD-L1 was significantly activated 12 hours after *S. aureus* infection. Unexpectedly, silencing of the* Slc7a11* gene using si-*Slc7a11* failed to block the expression of PD-L1 induced by *S. aureus*, but further augmented the mRNA expression of PD-L1 in BMDMs (Figure S6C). Moreover, immunofluorescence staining showed a further increase in PD-L1 protein levels in si-*Slc7a11*-treated BMDMs compared with those treated with si-NC (Figure S6D and S6E). In the next evaluation of the role of SLC7A11 in the expression of PD-L1 *in vivo*, we observed surprisingly no significant difference in the number of F4/80^+^PD-L1^+^ cells between *Lyz2Cre-Scl7a11^f/f^* mice and *Slc7a11^f/f^* mice without infection (Figure 6C and 6D), indicating that specific knockout of *Slc7a11* in macrophages failed to activate the expression of PD-L1 under non-infectious condition. In consistent with our previous study 16, the number of F4/80^+^PD-L1^+^ cells significantly increased in the* S. aureus*-infected bone of *Slc7a11^f/f^* mice compared with the non-infected ones (Figure 6C and 6D). Importantly, in mice model of osteomyelitis induced by *S. aureus*, the number of F4/80^+^PD-L1^+^ cells further increased in *Lyz2Cre-Scl7a11^f/f^* mice compared with *Slc7a11^f/f^* mice (Figure 6C and 6D). To confirm the role of SLC7A11 in regulation of PD-L1 expression after *S. aureus* infection, we isolated bone marrow cells from the *Lyz2Cre-Scl7a11^f/f^* mice and the *Slc7a11^f/f^* mice, differentiated them into BMDMs and then infected them with *S. aureus* for 12 hours. The results also revealed that macrophage-specific knockout of* Slc7a11* significantly activated PD-L1 expression only in the presence of *S. aureus* infection (Figure 6E and 6F). These findings suggest that activated SLC7A11 may restrict PD-L1 expression in macrophages following *S. aureus* infection.

NF-κB is a pivotal transcription factor for PD-L1 transcription by binding to its promoter 35-37, and ROS burst can activate the NF-κB pathway by phosphorylation modification of NF-κB functional subunits 38. Here, we found that *S. aureus* infection up-regulated the protein levels of p-NF-κB in BMDMs, and knockdown of SLC7A11 further promoted p-NF-κB levels (Figure 6G and 6H). Importantly, ROS scavengers such as NAC blocked the stimulatory effect of si-*Slc7a11* on p-NF-κB protein, while co-administration of ROS inducers like BSO and si-*Slc7a11* synergistically enhanced p-NF-κB protein levels (Figure 6G and 6H). Furthermore, EVP4539, a pharmacological inhibitor of NF-κB, markedly blocked the activation of PD-L1 signaling in BMDMs induced by si-*Slc7a11* treatment (Figure 6I and 6J). Together, these findings suggest that SLC7A11 may not contribute to regulating the expression PD-L1 under physiological condition due to the low basal level of ROS and the non-active NF-κB pathway. However, it plays an essential role in restricting the aberrant expression of PD-L1 via the ROS-NF-κB axis in response to persistent *S. aureus* infection.

### SLC7A11 deficiency enhances the sensitivity of anti-PD-L1 immunotherapy in mice with *S. aureus* osteomyelitis

**Figure 7:**
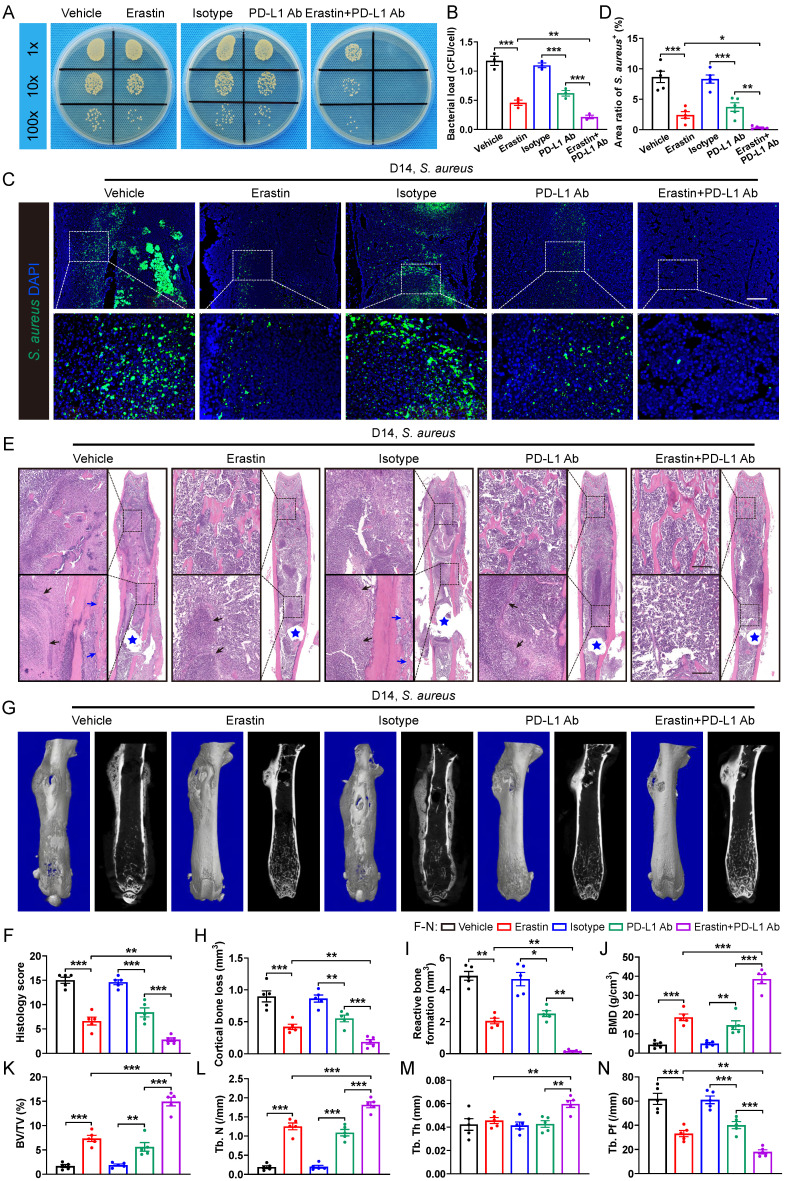
** SLC7A11 deficiency enhances the sensitivity of anti-PD-L1 immunotherapy in mice with *S. aureus* osteomyelitis. (A and B)** Representative images and quantification of bacterial burden in BMDMs at 12 hours of infection. Prior to *S. aureus* infection, BMDMs were pre-treated with erastin (5 µM) alone, or PD-L1 neutralizing antibody (PD-L1 Ab, 10 µg/mL) alone, or a combination of erastin (5 µM) and PD-L1 Ab (10 µg/mL) for one hour. n = 3/group. **(C and D)** Representative immunofluorescence images of *S. aureus* in the femurs from *S. aureus* osteomyelitis mice. Mice were randomly divided into 5 groups and treated with vehicle (10% DMSO in 0.9% saline), erastin (30 mg/kg of body weight), isotype antibody (200 µg/mice), PD-L1 Ab (200 µg/mice), or a combination of erastin (30 mg/kg of body weight) and PD-L1 Ab (200 µg/mice) once every two days. Quantification of the percentage of *S. aureus*^+^ areas out of total bone marrow area is shown in (D). Mice were euthanized and the right femurs were collected at day 14 after implant-associated *S. aureus* osteomyelitis surgery. Scale bar, 200 µm. n = 5/group. **(E and F)** Representative images of H&E staining and histological scores of the femurs from *S. aureus* osteomyelitis mice receiving indicated treatments. Blue stars show the position of the implant in bone marrow cavity. Dark arrows show abscess in bone marrow cavity, and blue arrows show reactive new bone formation. Scale bar, 200 µm. n = 5/group. **(G)** Representative images of three-dimensional and coronal micro-CT of the femurs from *S. aureus* osteomyelitis mice with indicated treatments. **(H and I)** Quantitative analysis of cortical bone loss and reactive bone formation of the femurs from *S. aureus* osteomyelitis mice with indicated treatments. n = 5/group. **(J-N)** Quantitative analysis of trabecular bone mineral density (BMD), bone fraction (BV/TV), trabecular number (Tb. N), trabecular thickness (Tb. Th) and trabecular bone pattern factor (Tb. Pf) of the femurs from *S. aureus* osteomyelitis mice with indicated treatments. Data are shown as means ± SEM. One-way ANOVA with Fisher's LSD *post hoc* test (B, D, F, H, J, K, L, M, N) or Dunnett's T3 *post hoc* test (I) was used. *P < 0.05, **P < 0.01, ***P < 0.001.

Next, we tested whether blocking SLC7A11 and anti-PD-L1 might have a synergistic effect on promoting antimicrobial activity of macrophages. Intracellular killing assay revealed that administration of either erastin or PD-L1 neutralizing antibodies (PD-L1 Ab) significantly enhanced the bactericidal activity of BMDMs* in vitro*. Notably, BMDMs treated with administration of both erastin and PD-L1 Ab exhibited a notable reduction in intracellular bacterial burden compared with those treated with either erastin or PD-L1 Ab alone (Figure 7A and 7B). In the mice model of *S. aureus*-induced osteomyelitis, a combined treatment with erastin and PD-L1 Ab led to a further reduced area of *S. aureus*^+^ regions compared with mice treated with erastin or PD-L1 Ab alone (Figure 7C and 7D). In consistent with the above findings, histopathological staining and scores showed a considerably improved bone structure and a significantly reduced abscess area within the bone marrow cavity in mice treated with a combination of erastin and PD-L1 Ab compared with the mice treated with either erastin or PD-L1 Ab alone (Figure 7E and 7F). Additionally, micro-CT data confirmed that combination of erastin and PD-L1 Ab had a synergistic effect on protecting mice against *S. aureus*-induced bone destruction, manifested by strikingly reduced cortical bone loss and less reactive bone formation, and notably improved bone density and trabecular microarchitecture (Figure 7G-7N). The above findings demonstrate that blocking both SLC7A11 and PD-L1 may have a synergistic effect to promote the bactericidal function of macrophages.

## Discussion

Survival within macrophages is one of the major strategies for* S. aureus* to evade immune response and resist antibiotics, ultimately leading to dissemination of *S. aureus* and persistent infection. However, it remains poorly characterized how *S. aureus* impairs antimicrobial activity of macrophages in the pathogenesis of osteomyelitis. The present study has identified critical roles of SLC7A11 activation in destroying the antimicrobial function of macrophages after persistent* S. aureus* infection. We demonstrated that pharmacological inhibition or genetic knockout of SLC7A11 can promote bactericidal function of macrophages, reduce bacterial burden in the bone and improve bone structure of mice with *S. aureus* osteomyelitis. Moreover, our data show that aberrantly expressed SLC7A11 in macrophages may down-regulate the level of intracellular ROS and reduce lipid peroxidation, contributing to the impaired bactericidal function of macrophages. It is interesting to note that inhibition of SLC7A11 may further activate expression of PD-L1, and a combination therapy of blocking SLC7A11 and PD-L1 facilitates clearance of* S. aureus in vitro* and *in vivo*. On the basis of our recent study on PD-L1 and the present study, we have further identified SLC7A11 as another important therapeutic target in macrophages for prevention of persistent infection in a setting of subacute *S. aureus* osteomyelitis.

The current study has extended our understanding of SLC7A11 in regulating ROS in bone marrow macrophages during *S. aureus* infection. It is well known that SLC7A11, as a critical catalytic subunit of the cystine/glutamate antiporter, imports cysteine for glutathione biosynthesis and thereby detoxifies ROS and maintains the redox balance which defends against oxidative stress in many solid tumor cells 21, 23, 39. Although recent study has addressed the critical role of SLC7A11 in increasing host susceptibility to *Plasmodium* or* Mycobacterium tuberculosis* by inhibiting ROS generation and lipid peroxidation 26, 27, our *in vivo* and* in vitro* data further demonstrated that aberrant expression of SLC7A11 may impair bactericidal activity by suppressing ROS production in macrophages. Given the intracellular persistence of *S. aureus* within macrophages during osteomyelitis pathogenesis, SLC7A11 overexpression may render cells more resistant to oxidative stress induced by intracellular bacteria, thus protecting cells from death and facilitating *S. aureus* persistence. Notably, flow cytometry data also revealed a substantial increase in SLC7A11 expression in neutrophils at day 14 post-infection, warranting further investigation into SLC7A11's role in regulating neutrophils function during *S. aureus* infection.

Our recent* in vitro* data have shown that PD-L1 expression is activated in BMDMs as early as 2 hours after* S. aureus* infection 16, but the present study has found that robust expression of SLC7A11 appears after 12 hours of infection, it is reasonable to postulate that these two molecules might play distinct roles in initiation and potentiation of the suppressive effect on ROS levels, respectively, in the context of persistent* S. aureus* infection. Recent studies have highlighted PD-L1 as a negative regulator of M1 polarization and the secretion of proinflammatory cytokines in macrophages, processes associated with ROS production and NF-κB pathway activation 40, 41. While we did not assess the effect of SLC7A11 and PD-L1 on macrophage polarization, both our present and previous work 16 have demonstrated their inhibitory effects on ROS production and the NF-κB pathway in *S. aureus*-infected BMDMs. Additionally, we have shown that activated PD-L1 expression may impair the bactericidal function of bone marrow macrophages by stimulating mitophagy, thereby reducing the level of ROS 16. In light of the findings from our present study, it is plausible to suggest that both SLC7A11 and PD-L1 impair macrophage bactericidal function at least partially via suppressed ROS levels.

The discovery of the inhibitory effect of SLC7A11 on PD-L1 expression in macrophages in the context of *S. aureus* infection may have practical implications. Indeed, the activated expression of PD-L1 by either knocking down or overexpression of* Slc7a11* has been determined in tumor cells or tumor associated macrophages in tumor models 24, 42, 43. However, little is known about the relationship between SLC7A11 and PD-L1 in *S. aureus* infection. Several studies have demonstrated the critical role of NF-κB/p65 in PD-L1 transcription and expression in tumor cells 35-37. Here, we found that inhibiting SLC7A11 further activates the expression of PD-L1 via the ROS-NF-κB/p65 axis following persistent *S. aureus* infection. It is worth noting that blocking SLC7A11 in macrophages does not alter PD-L1 expression under physiological conditions in our work. This may be attributed to the low basal levels of NF-κB and ROS observed in our study, as well as the low expression level of SLC7A11, given its role as a stress-responsive molecule 21, 23. Previous research, including ours, has demonstrated that abnormal activation of PD-L1 impedes the immune defense mechanisms, like driving T cell exhaustion and weakening the bactericidal ability of hepatic sinusoidal Kupffer cells or peritoneal macrophages 16, 44-46. Here, we have found that blocking both SLC7A11 and PD-L1 has a synergistic effect on activating intracellular ROS to improve bactericidal function of macrophages, suggesting that the roles of SLC7A11 and PD-L1 signaling may be overlapping and distinct in regulation of antimicrobial activities of macrophages.

This study highlights the potential application of erastin as a reagent that may potentiate innate immunity during persistent *S. aureus* infection. Erastin and its analogs are known as inducers of ferroptosis, a type of cell death characterized by accumulation of intracellular lipid peroxides and ROS, by inhibiting SLC7A11-mediated cystine uptake and activating the voltage-dependent anion channels (VDACs) to induce mitochondrial dysfunction in tumor cells 47-49. Our studies have advanced these investigations that erastin increases bactericidal function of bone marrow macrophages by improving ROS production during persistent *S. aureus* infection. However, it is important to note that there are possible side effects of erastin* in vivo* due to the ubiquitous expression of SLC7A11 and VDACs in various tissues. The toxicity of erastin and its analogs seems to depend on the intervals of treatment. Indeed, erastin injection at a dose of 25 mg/kg twice daily may induce iron deposition and pathological changes in multiple organs after 2 days of treatment in healthy mice 50, while erastin and its analogs have promising effects on restricting tumor growth with minimal toxic and side effects at dose of 30-50 mg/kg once daily or once every other day for 14 days 51, 52. Therefore, the dosage of erastin (30 mg/kg, once every other day) we used in the present study was effective and safe for short-term control of *S. aureus* infection. However, future studies are needed to further assess the potential long-term side effects of erastin.

Although the immune activation mediated by SLC7A11 blockade has shown a promising therapeutic potential for *S. aureus*-induced osteomyelitis, there are still several aspects that warrant further investigation. Firstly, as transition of macrophage immunosuppressive states is accompanied by metabolic reprogramming, such as attenuated aerobic glycolysis and enhanced oxidative phosphorylation 53, it needs further elucidation whether or not the decreased antibacterial activity of macrophages following persistent *S. aureus* infection may involve the metabolic reprogramming in the macrophages mediated by SLC7A11. Secondly, now that other pathogenic bacteria besides *Staphylococcus aureus* participate in the pathogenesis of implant-associated infectious osteomyelitis, including *Pseudomonas aeruginosa*, *Staphylococcus epidermidis*, *Escherichia coli*, and *Enterococcus faecalis* 1, 4, 6, it is still unclear whether SLC7A11 can be activated or not by these pathogens thereby exerting suppressive effects on immune responses in macrophages.

In conclusion, this study identifies abnormally activated SLC7A11 expression in macrophages as a critical mediator responsible for the impaired antimicrobial activity in a mice model of *S. aureus*-induced osteomyelitis, and also reveals that SLC7A11 has a suppressive effect on the PD-L1 signaling in persistent* S. aureus* infection. Therefore, targeting both SLC7A11 and PD-L1 may be a promising immunomodulatory strategy for treatment of *S. aureus* osteomyelitis at a subacute or chronic stage.

## Materials and Methods

### Bacterial culture and quantification

The wild-type *S. aureus* strain used in this study was isolated from an individual with infectious osteomyelitis. Using a sterile inoculating loop, the monoclonal *S. aureus* was transferred into fresh tryptic soy broth (TSB), and was subsequently cultured at 37°C in a bacterial incubator with a rotation speed of 180 rpm for 16-18 hours. After washing twice with phosphate buffered saline (PBS), the optical density of the bacterial solution was adjusted to 0.5 at a wavelength of 600 nm. Under this condition, the bacterial density is approximately 1×10^8^ colony forming units per milliliter (CFU/mL).

### Mouse models

All animal experiments and procedures were conducted in accordance with the ARRIVE Guidelines 2.0 54 and approved by the Animal Welfare and Use Committee of Nanfang Hospital, Southern Medical University. Wild-type C57BL/6 mice were purchased from the Experimental Animal Center of Southern Medical University. *Lyz2Cre* mice (C57BL/6 background) were from the Jackson Laboratory (Stock No. 004781). *Rosa^tdTomato^* reporter mice (C57BL/6 background) were from the Jackson Laboratory (Stock No. 007914). *Slc7a11^flox/flox^* mice (C57BL/6 background) were obtained from GemPharmatech Co., Ltd (Jiangsu, China). PCR analysis was performed on genomic DNA extracted from mouse tail fragments using the following primers to determine the genotypes of newborn mice: *Lyz2Cre* mutant, 5'-CCCAGAAATGCCAGATTACG-3', *Lyz2Cre* wild type, 5'-TTACAGTCGGCCAGGCTGAC-3', and *Lyz2Cre* common, 5'-CTTGGGCTGCCAGAATTTCTC-3'; *Rosa^tdTomato^* mutant forward, 5'-CTGTTCCTGTACGGCATGG-3', *Rosa^tdTomato^* mutant reverse, 5'-GGCATTAAAGCAGCGTATCC-3', *Rosa^tdTomato^* wild type forward, 5'-AAGGGAGCTGCAGTGGAGTA-3', and *Rosa^tdTomato^* wild type reverse, 5'-CCGAAAATCTGTGGGAAGTC-3'; loxP-flanked SLC7A11 allele forward, 5'-GAAGATGAGTCTAGGGTGTGGTCTTC-3', and SLC7A11 reverse, 5'-GACTGACACAGCTAACACACCACATG-3'. All mice were housed in a standard specific pathogen-free facility, and were provided with ad libitum access to normal water and food.

To establish an animal model of implant-associated *S. aureus* infectious osteomyelitis, the following procedure was conducted using the previously described methods 28. Briefly, mice (8-12 weeks old) of both sexes were anesthetized with intraperitoneal injection of tribromoethanol (125 mg/kg of body weight), and then ophthalmic forceps were used to bluntly separate the muscle and expose the third trochanter of the right femur. Subsequently, a unicortical hole was drilled out using a 27-gauge needle. *S. aureus* suspension (2×10^5^ CFU/mL, 3 µl) or an equal volume of PBS was slowly injected into the marrow cavity using a microliter syringe to establish *S. aureus*-infected mice and control mice, respectively. After injection, a sterile self-tapping screw was drilled into the cortical bone through the hole. Finally, the surgical incision was sutured using 5-0 sutures. All mice received daily intraperitoneal injections of gentamicin (20 mg/kg of body weight) for postoperative treatment. Additionally, to investigate the role of SLC7A11 and PD-L1 signaling in the pathogenesis of *S. aureus* osteomyelitis, mice were subjected to erastin treatment (30 mg/kg of body weight; MedChemExpress, USA) or a combination with PD-L1 Ab (200 µg/mouse, Bio X cell, USA) through intraperitoneal injection. The treatments were initiated on the first day post-surgery and administered every other day for a duration of 2 weeks.

### Histological analysis

Paraffin-embedded femurs were sectioned coronally at 4-µm thickness and stained with hematoxylin and eosin (H&E) according to the manufacturer's protocol (Beyotime, China). In brief, after deparaffinization and rehydration, sections were stained with hematoxylin for 3 minutes, differentiated in 1% hydrochloric acid ethanol for 2 seconds, and stained with eosin for 30 seconds. For evaluation of histopathological changes of bones, we modified Smeltzer's scoring system 55 based on histologic features of “acute” and “chronic” osteomyelitis in osteomyelitis patients 7 and our findings in mice model of osteomyelitis 28. Briefly, the score for each sample was a sum of the individual sub-scores of 4 parameters including intraosseous acute inflammation (small focus of neutrophils with absence or presence of intramedullary abscess), intraosseous chronic inflammation (large and/or multiple foci of neutrophils with absence or presence of intramedullary fibrosis), periosteal reaction (periosteal inflammation with absence or presence of subperiosteal abscess formation), and bone necrosis (necrosis with absence or presence of sequestra). Each parameter was scored with 0 points for “normal” up to 4 points for “severe”.

### Immunofluorescence and immunohistochemistry

Immunofluorescence staining was performed according to standard protocols. Briefly, right femurs of the mice were fixed in 4% paraformaldehyde solution overnight, decalcified in 0.5 M ethylenediaminetetraacetic acid (EDTA) solution (pH = 8.0) for 7 days with change of solution every 2 days. After being infiltrated with 30% sucrose overnight at 4°C, femurs were embedded in optimal cutting temperature (OCT) medium and kept at -80°C until sectioning. Frozen sections at 15-µm thickness were thawed at 37°C for 20 minutes, and then washed with 0.1% PBST and PBS sequentially. After being blocked in 10% goat serum in PBST at room temperature for 1 hour, the sections were incubated with primary antibodies overnight at 4°C. The primary antibodies used in this study were as follows: *S. aureus* (1:200, PA1-7246, Invitrogen), SLC7A11 (1:500, ab307601, Abcam), PD-L1 (1:200, ab279292, Abcam), and F4/80 (1:100, AS-MCA497GA, Bio-Rad). The next day, the sections were incubated with fluorescence-conjugated secondary antibodies at room temperature for 1 hour and mounted with an anti-fluorescence quenching reagent containing 4',6-diamidino-2-phenylindole (DAPI). Representative images were obtained using a Zeiss LSM980 laser confocal microscope. The secondary antibodies included Alexa Fluor 488-conjugated goat anti-rabbit IgG (1:500, #4412, Cell signaling Technology), Alexa Fluor 594-conjugated goat anti-mouse IgG (1:400, #8890, Cell signaling Technology), and Dylight 488-conjugated goat anti-rat IgG (1:200, A23240, Abbkine). To assess the bacterial load in marrow macrophages *in vivo*, three random field areas at low magnification surrounding the femoral nail or abscess of each mouse were selected for photography. The area of double-positive staining for *Lyz2* and *S. aureus* in each field was quantified using Image J software (FIJI version, NIH, USA) and expressed as a ratio to the total area of marrow tissue (DAPI-positive staining area). The average ratio was then calculated. Each group comprised at least 5 mice.

For immunohistochemistry staining, the 4-µm paraffin sections were subjected to antigen retrieval with 10 mM EDTA (pH = 9.0) and quenching endogenous peroxidase activity with 3% hydrogen peroxide. After that, the sections were blocked with 10% goat serum at room temperature for 1 hour, and then incubated with a specific primary antibody to SLC7A11 (1:300, ab307601, Abcam) at 4°C overnight, followed by incubation with an HRP-conjugated secondary antibody (1:200, HA1001, Huabio) at room temperature for 1 hour. Finally, the sections were stained with a 3,3'-Diaminobenzidine (DAB) Kit (ZLI-9018, ZSGB-BIO, China) to detect the peroxidase signal and counterstained with hematoxylin.

### Flow sorting

After 14 days of infection, bone marrow cells from the right femur of mice were flushed out using a 27-gauge syringe and filtered through a 70-µm tissue sieve. Red blood cells were removed from the single cell suspension using ammonium-chloride potassium (ACK) lysis buffer. The cell suspension was then incubated on ice for 15 minutes with anti-mouse CD16/32 antibody (1 µg/10^6^ cells, #101319, Biolegend) to block non-specific endogenous signals. Subsequently, cells were incubated with anti-mouse CD11b-BV421 (1 µg/10^6^ cells, #101235, Biolegend) and anti-mouse F4/80-APC/Fire 750 (0.5 µg/10^6^ cells, #123152, Biolegend) antibodies on ice in the dark for 30 minutes. After being washed twice with 0.1% bovine serum albumin (BSA) solution in PBS, CD11b^+^F4/80^+^ macrophages were sorted using a BD FACS Aria II flow cytometer (BD Bioscience, USA).

### High-throughput RNA sequencing and tandem mass tags (TMT)-based quantitative proteomics

On day 14 post-surgery, the CD11b^+^F4/80^+^ macrophages sorted from *S. aureus*-infected mice (n = 3) and control mice (n = 3) were used for high-throughput RNA sequencing analysis and TMT-based proteomics analysis. For high-throughput RNA sequencing, after extracting the total RNA of CD11b^+^F4/80^+^ cells using Trizol reagent, the quality of RNA samples was evaluated by agarose gel electrophoresis and Agilent 2100 Bioanalyzer. Subsequently, eukaryotic mRNA was enriched using magnetic beads with Oligo (dT), and the enriched nucleic acid samples were fragmented. After sequential steps of reverse transcription, cDNA fragment end modification, and addition of single “A” base, fragments of approximately 200 bp were selected using AMPure XP beads and amplified by polymerase chain reaction (PCR). The cDNA library sequencing was conducted by Gene Denovo Biotechnology Co., Ltd (Guangzhou, China) using the Illumina Novaseq6000 sequencer. The read count data of gene expression in the sequencing library were normalized and subjected to differential expression analysis using the DESeq2 R package 56. Genes that meet the criteria of false discovery rate (FDR) less than 0.05 and absolute fold change equal to or greater than 2 were considered as DEGs.

For TMT-based proteomics analysis, dithiothreitol and iodoacetamide were used to break disulfide bonds and initiate reductive alkylation on the proteins extracted from the CD11b^+^F4/80^+^ cells. Subsequently, the protein was digested using trypsin buffer at 37°C for 2 hours, and the peptide mixtures were labeled with isobaric tag for relative and absolute quantification (iTRAQ)/TMT labeling reagents (Thermo Fisher, USA). Labeled peptides were then transferred to a reverse-phase spin column and subjected to high pH reverse-phase separation. The fractionated components were analyzed using low-pH nano-HPLC-MS/MS (Orbitrap Fusion) liquid chromatography-mass spectrometry (Thermo Fisher, USA) by Gene Denovo Biotechnology Co., Ltd (Guangzhou, China), with data acquisition mode set to data-dependent acquisition (DDA). DEGs in the translatomes were defined as a *P-value* less than 0.05 and an absolute fold change greater than or equal to 2.

### GO enrichment analysis and GSEA

The cluster Profiler package (4.8.1) was used for GO functional enrichment analysis and GSEA analysis of transcriptomic data. For GO analysis, the ggplot2 package (4.3.0) was used to visualize the enriched biological processes. GO items with adjusted *P-values* less than 0.05 were considered significantly enriched. Additionally, the GseaVis package (0.0.8) and enrichplot package (1.20.0) were used for GSEA analysis and data visualization. The significance cutoff values were defined as follows: absolute Normalized Enrichment Score (NES) ≥ 1, *P-value* < 0.05, Benjamini-Hochberg false discovery rate < 0.25.

### Mouse BMDMs culture and intracellular ROS detection

Primary mouse BMDMs were isolated and cultured as described previously 16. Briefly, bone marrow cells were flushed out and cultured in RPMI-1640 medium supplemented with 30% L929 fibroblast cell-conditioned medium and 1% penicillin/streptomycin. On day 7, mature BMDMs were stimulated with *S. aureus* bacterial solution at a MOI of 10 for 1 hour, followed by an additional one hour of culture in fresh medium containing lysostaphin (20 µg/mL) and gentamicin (50 µg/mL) to lyse extracellular bacteria. Thereafter, cells were cultured in fresh medium for 0, 6, 12 or 24 hours. At the indicated time points, the intracellular ROS levels were detected using the DCFH-DA probe (Beyotime, China). Briefly, cells were washed twice with serum-free medium, followed by incubation with a staining solution containing 10 µM DCFH-DA and 10 µg/mL Hoechst at 37°C in the dark for 20 minutes. Subsequently, the cells were washed three times with serum-free medium and immediately detected using a laser confocal microscopy (Zeiss LSM980, Germany) or a BD LSRII flow cytometer (BD Biosciences, USA).

### Real-time quantitative polymerase chain reaction (RT-qPCR)

The total cellular RNA was extracted using the RNAiso PLUS reagent. According to the manufacturer's instructions, reverse transcription and quantitative PCR were performed using the Evo M-MLV RT Premix Kit (Accurate Biology, China) and SYBR Green Premix Pro Taq HS qPCR Kit (Accurate Biology, China) on the Applied Biosystems QuantStudio 5 system. The *Actb* gene was used as the internal reference, and the relative gene expression was calculated using the 2^-∆∆CT^ method. The primer sequences for qPCR are as follows:* Slc7a11* forward, 5'-CAGGCATCTTCATCTCCCCC-3', and *Slc7a11* reverse, 5'-GCCAGCAAAGGACCAAAGAC-3'; *Pd-l1* forward, 5'-GCTCCAAAGGACTTGTACGTG-3', and *Pd-l1* reverse, 5'-TGATCTGAAGGGCAGCATTTC-3'; *Actb* forward, 5'-GCTTCTTTGCAGCTCCTTCGTT-3', and *Actb* reverse, 5'-CGGAGCCGTTGTCGACGACC-3'; *TNF-ɑ* forward, 5'-TCTCATGCACCACCATCAAGGACT-3', and *TNF-ɑ* reverse, 5'-ACCACTCTCCCTTTGCAGAACTCA-3'; *IL-1β* forward, 5'-TCCTGTGTAATGAAAGACGGC-3', and *IL-1β* reverse, 5'-ACTCCACTTTGCTCTTGACTTC-3'; *IL-6* forward, 5'-CCCCAATTTCCAATGCTCTCC-3', and *IL-6* reverse, 5'-CGCACTAGGTTTGCCGAGTA-3'.

### Flow cytometry

The implant-associated osteomyelitis mice model was established following the aforementioned procedures. *S. aureus*-infected mice and control mice were euthanized on postoperative days 3, 7, and 14. Red blood cells were removed from femoral marrow cells using ACK lysis buffer. After blocking with anti-mouse CD16/32 antibody (1 µg/10^6^ cells, #101319, Biolegend) on ice for 15 minutes, the cell suspension was incubated with a combination of mouse-specific antibodies in the dark for 40 minutes. This cocktail included anti-CD11b-FITC (0.25 µg/10^6^ cells, #101205, Biolegend), anti-F4/80-APC/Fire 750 (0.5 µg/10^6^ cells, #123151, Biolegend), anti-Ly6G-BV510 (0.5 µg/10^6^ cells, #127633, Biolegend), anti-Ly6C-PerCP (0.25 µg/10^6^ cells, #128027, Biolegend), and anti-SLC7A11 (1:100, ab307601, Abcam). Following this, the cell suspension was incubated on ice for 40 minutes with Alexa Fluor 647-conjugated goat anti-rabbit IgG secondary antibody (1:100, #4414, Cell signaling Technology). After two washes with PBS containing 0.1% BSA, the cells were immediately analyzed using a BD LSRII flow cytometer (BD Biosciences, USA). FlowJo software (version 10, FlowJo, USA) was used to assess the expression levels of SLC7A11 in CD11b^+^F4/80^+^ macrophages, CD11b^+^Ly6G^+^ neutrophils, and CD11b^+^Ly6C^+^ monocytes.

### Western blot

In brief, primary BMDMs were lysed in ice-cold RIPA buffer containing 1% phosphatase inhibitors and 1% protease inhibitors. Protein concentration was determined using the BCA Protein Assay Kit (Beyotime, China). Equal amounts of protein (20-30 µg) were separated by SDS-PAGE and transferred to a PVDF membrane by electrophoresis. After blocking with 5% skim milk at room temperature for 1.5 hours, the PVDF membrane was incubated with primary antibodies against SLC7A11 (1:1000, 26864-1-AP, Proteintech), β-Actin (1:20000, 66009-1-Ig, Proteintech), p-NF-κB (1:2000, AF2006, Affinity), NF-κB (1:2000, AF5006, Affinity), and PD-L1 (1:1000, 17952-1-AP, Proteintech) overnight at 4°C, followed by incubation with horseradish peroxidase-conjugated secondary antibodies at room temperature for 1 hour. Protein bands were visualized using a chemiluminescence imaging system (BLT GelView 6000 Pro, China) after thoroughly wetting the PVDF membrane with electrochemiluminescence reagent.

### RNA interference

Specific small interfering RNA (siRNA) targeting *Slc7a11* (si-*Slc7a11*) and its corresponding negative control (si-NC) were chemically synthesized by Sangon Biotech Co., Ltd (Shanghai, China). According to the manufacturer's instructions, EndoFectin reagent (Genecopoeia, USA) was used for cell transfection. Briefly, by day 6 after seeding, the culture medium of primary BMDMs was replaced with fresh medium without antibiotics. After 24 hours, the medium was replaced with Opti-MEM medium. The transfection reagent and siRNA were gently mixed and incubated at room temperature for 20 minutes before being added to the Opti-MEM medium. After 9 hours of transfection, the medium was replaced by RPMI-1640 medium containing 30% L929 fibroblast cell-conditioned medium, and the cells were cultured for an additional 48 hours before conducting subsequent experiments. The siRNA sequences were as follows: si-*Slc7a11*, 5'-TGGGTGGAACTGCTCGTAATA-3'; si-NC, 5'-UUCUCCGAACGUGUCACGUTT-3'.

### Intracellular killing assay

Primary BMDMs were seeded at a density of 3×10^5^ cells/well in a 24-well plate. By day 7, after being pre-treated with 5 µM erastin or vehicle (DMSO) for 1 hour, BMDMs were infected with *S. aureus* at a MOI of 10 for 1 hour. Then, cells were incubated with medium containing lysostaphin (20 µg/mL) and gentamicin (50 µg/mL) to lyse the non-phagocytosed extracellular bacteria. After being washed twice with PBS, cells were then cultured in medium with the presence or absence of 5 µM erastin for an extra 12 hours. Finally, the cells were lysed with 0.1% Triton X-100 in PBS at room temperature for 15 minutes, and the bacterial suspension was serially diluted and cultured on TSB agar plates for 16-18 hours. The colony count was used to determine the bactericidal activity of BMDMs, with bacterial load defined as the average number of *S. aureus* within each BMDM.

### Lipid peroxidation

The Image-iT Lipid Peroxidation Assay Kit (Thermo Fisher, USA) was used to detect the levels of lipid peroxidation in live cells. Briefly, primary BMDMs were seeded in confocal dishes at a density of 4×10^5^ cells/dish and cultured for 7 days. After infection with *S. aureus* with the presence or absence of reagent treatments as indicated, the cells were incubated at 37°C for 30 minutes in a serum-free staining solution containing 10 µM C11-BODIPY (581/591) and 10 µg/mL Hoechst. After three washes with PBS, cells were observed and imaged using a Zeiss LSM980 laser confocal microscope. According to the manufacturer's instructions, the excitation/emission wavelengths were 581/590 nm (red fluorescence) for reduced state lipids, and 488/510 nm (green fluorescence) for oxidized state lipids.

The malondialdehyde (MDA) Colorimetric Assay Kit (Elabscience, China) was used for quantitative analysis of the lipid peroxidation marker MDA 33. Approximately 2×10^6^ mature BMDMs or CD11b^+^F4/80^+^ cells were transferred to a 1.5 mL centrifuge tube, and 0.5 mL of extracting solution was added. The cells were thoroughly lysed using an ultrasonic disruptor. After homogenizing the cell suspension, 0.1 mL of the suspension was taken for protein concentration measurement using the BCA method. Another 0.1 mL of the suspension was transferred to a new centrifuge tube labeled as the sample tube. Following the manufacturer's protocol, the working solution was prepared by mixing the clarificant, acid regent, and chromogenic agent at a volume ratio of 1:15:5. Then, 1 mL of the working solution was added to the sample tube and incubated in a water bath at 100°C for 40 minutes. After centrifugation at 1078 rcf for 10 minutes, 0.25 mL of the supernatant was transferred to a 96-well plate and the absorbance value at 532 nm wavelength was measured using a microplate reader (SpectraMax i3X, USA).

### Micro-computed tomography (micro-CT) analysis

After 14 days of infection, right femurs of the mice were scanned using high-resolution micro-CT (SkyScan1276, Bruker, Germany). The voxel size of all images was 6 µm, with a current of 145 mA, a voltage of 55 kV, and an integration time of 400 ms. The three-dimensional structural parameters of the cortical bone and trabecular microstructure parameters were analyzed with previously described methods 16. Bone mineral density (BMD) was calibrated using ceramic standard samples. Initially, the original scan data were reconstructed into two-dimensional images using NRecon software (version 1.6.8, Bruker, Germany). Then, the DataViewer software (version 1.5.4, Bruker, Germany) was used to recalibrate the position of femurs based on their images in the coronal, sagittal, and transaxial planes. Subsequently, CTAn software (version 1.9, Bruker, Germany) was employed to analyze bone structure parameters in selected regions of interest (ROI) on each plane, with data from all planes recorded and overlaid. For the three-dimensional structural parameters of cortical bones, such as cortical bone loss and reactive bone formation, the ROI spanned 1200 layers (7.2 mm) centered around the monocortical hole of the self-tapping screw. In addition, trabecular microstructure assessment extended longitudinally from 0.444 mm away from the growth plate to a proximal distance of 1.2 mm. Trabecular microstructure parameters comprised bone mineral density (BMD), ratio of bone volume to total volume (BV/TV), trabecular number (Tb. N), trabecular thickness (Tb. Th), and trabecular bone pattern factor (Tb. Pf). Finally, CTVol software (version 2.0, Bruker, Germany) was used to visualize the three-dimensional bone models and generate representative images. All procedures for sample scanning and data analysis strictly adhere to guidelines for assessing bone structures in rodents using micro-CT 57.

### Statistical analysis

All data were presented as means ± standard errors of the mean (SEM). Statistical analysis was performed using IBM SPSS Statistics 20. Normality was checked via Shapiro-Wilk test. For comparisons between two groups, an unpaired two-tailed Student's t-test was used. For multiple comparisons, one-way or two-way ANOVA with Fisher's LSD *post hoc* test or Dunnett's T3 *post hoc* test was used. A *p-value* less than 0.05 was considered statistically significant.

## Supplementary Material

**Figure S1.** Pathogenesis of *S. aureus*-induced osteomyelitis is accompanied by an immunosuppressive state in macrophages. **Figure S2.** SLC7A11 expression is up-regulated in macrophages after *S. aureus* infection. **Figure S3.** Inhibition of SLC7A11 enhances the bactericidal capacity of macrophages by inducing ROS generation and lipid peroxidation. **Figure S4.** Erastin treatment ameliorates the pathogenesis of *S. aureus* osteomyelitis in mice. **Figure S5.** Macrophage-specific knockout of *Slc7a11* ameliorates the pathogenesis of *S. aureus* osteomyelitis in mice. **Figure S6.** Blocking SLC7A11 promotes PD-L1 expression via the ROS-NF-κB axis in macrophages after *S. aureus* infection.
