# Supplementary material for: Aberrant Expression of SLC7A11 Impairs the Antimicrobial Activities of Macrophages in Staphylococcus Aureus Osteomyelitis in Mice
Source: Int J Biol Sci. 2024 Apr 22;20(7):2555–75. doi: 10.7150/ijbs.93592 (PMC11077379; doi:10.7150/ijbs.93592)
Supplement: Supplementary file 1 — Figure S1. Pathogenesis of S. aureus-induced osteomyelitis is accompanied by an immunosuppressive state in macrophages. Figure S2. SLC7A11 expression is up-regulated in macrophages after S. aureus infection. Figure S3. Inhibition of SLC7A11 enhances the bactericidal capacity of macrophages by inducing ROS generation and lipid peroxidation. Figure S4. Erastin treatment ameliorates the pathogenesis of S. aureus osteomyelitis in mice. Figure S5. Macrophage-specific knockout of Slc7a11 ameliorates the pathogenesis of S. aureus osteomyelitis in mice. Figure S6. Blocking SLC7A11 promotes PD-L1 expression via the ROS-NF-κB axis in macrophages after S. aureus infection. [file ijbsv20p2555s1.pdf]

Aberrant Expression of SLC7A11 Impairs the Antimicrobial Activities of Macrophages in *Staphylococcus Aureus* Osteomyelitis in Mice

Bingsheng Yang[1, 2]†, Wen Shu[1, 2, 3]†, Jin Hu[1, 2], Zhongwen Wang[1, 2], Jichang Wu[1, 2], Jianwen Su[1, 2], Jianye Tan[4], Bin Yu[1, 2]*, Xianrong Zhang[1, 2]*

[1] Division of Orthopaedics and Traumatology, Department of Orthopaedics, Nanfang Hospital, Southern Medical University.

[2] Guangdong Provincial Key Laboratory of Bone and Cartilage Regenerative Medicine, Nanfang Hospital, Southern Medical University.

[3] Department of Trauma Orthopedics, Liuzhou People's Hospital.

[4] Department of Orthopedics, The Second Affiliated Hospital of Nanchang University.

† Bingsheng Yang and Wen Shu contributed equally to this work.

* Corresponding authors:

Xianrong Zhang, Division of Orthopaedics and Traumatology, Department of Orthopaedics, Nanfang Hospital, Southern Medical University, No.1838 North of Guangzhou Avenue, Guangzhou, Guangdong Province, China, 510515. Email: xianrongzh@smu.edu.cn, ORCID: 0000-0002-0992-5013. Tel.: 86-020-61641744.

Bin Yu, Division of Orthopaedics and Traumatology, Department of Orthopaedics, Nanfang Hospital, Southern Medical University, No.1838 North of Guangzhou Avenue, Guangzhou, Guangdong Province, China, 510515. Email: yubinol1@163.com. Tel.: 86-020-61641741.

Supplementary Figures

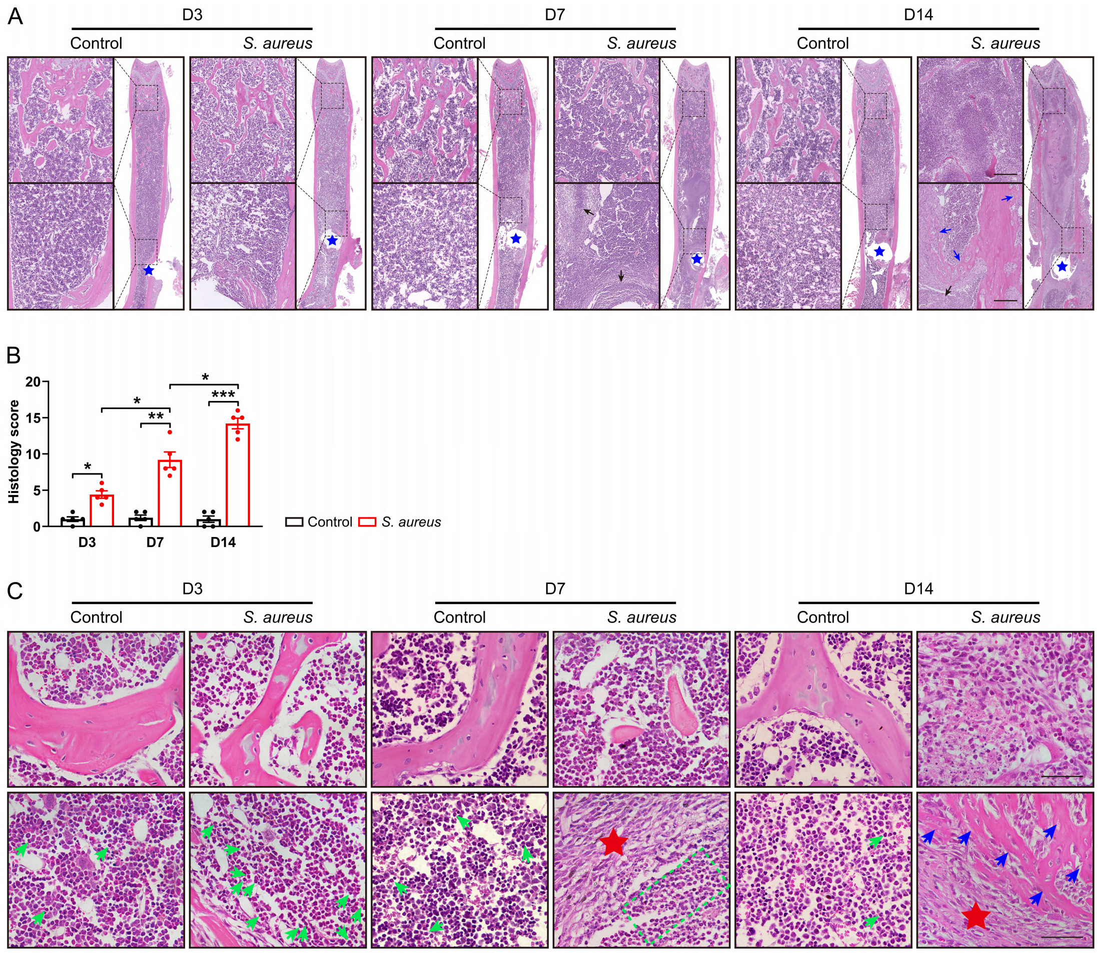

Figure S1. Pathogenesis of *S. aureus*-induced osteomyelitis is accompanied by an immunosuppressive state in macrophages. (A and B) Representative images of H&E staining and histological scores of the femurs from the mice model of implant-associated *S. aureus* osteomyelitis and control ones. Blue stars show the position of the implant in bone marrow cavity. Dark arrows show abscess in bone marrow cavity, and blue arrows show reactive new bone formation. D3, D7, and D14 represent time points of sample collection by days 3, 7, and 14 after surgery, respectively. Scale bar, 200 μm. n = 5/group. **(C)** Representative images of H&E staining of the femurs from the mice model of implant-associated *S. aureus* osteomyelitis and control ones. The upper and lower images respectively illustrate the medullary cavity structure around the metaphyseal trabecular bone and implantation site at high magnification. Green arrows show neutrophils, red stars show fibrosis,

green dotted box shows infiltration of neutrophils, and blue arrows show reactive new bone formation. D3, D7, and D14 represent time points of sample collection by days 3, 7, and 14 after surgery, respectively. Scale bar, 100 μm. Data are shown as means ± SEM. One-way ANOVA with Dunnett's T3 *post hoc* test was used. *$P < 0.05$, **$P < 0.01$, ***$P < 0.001$.

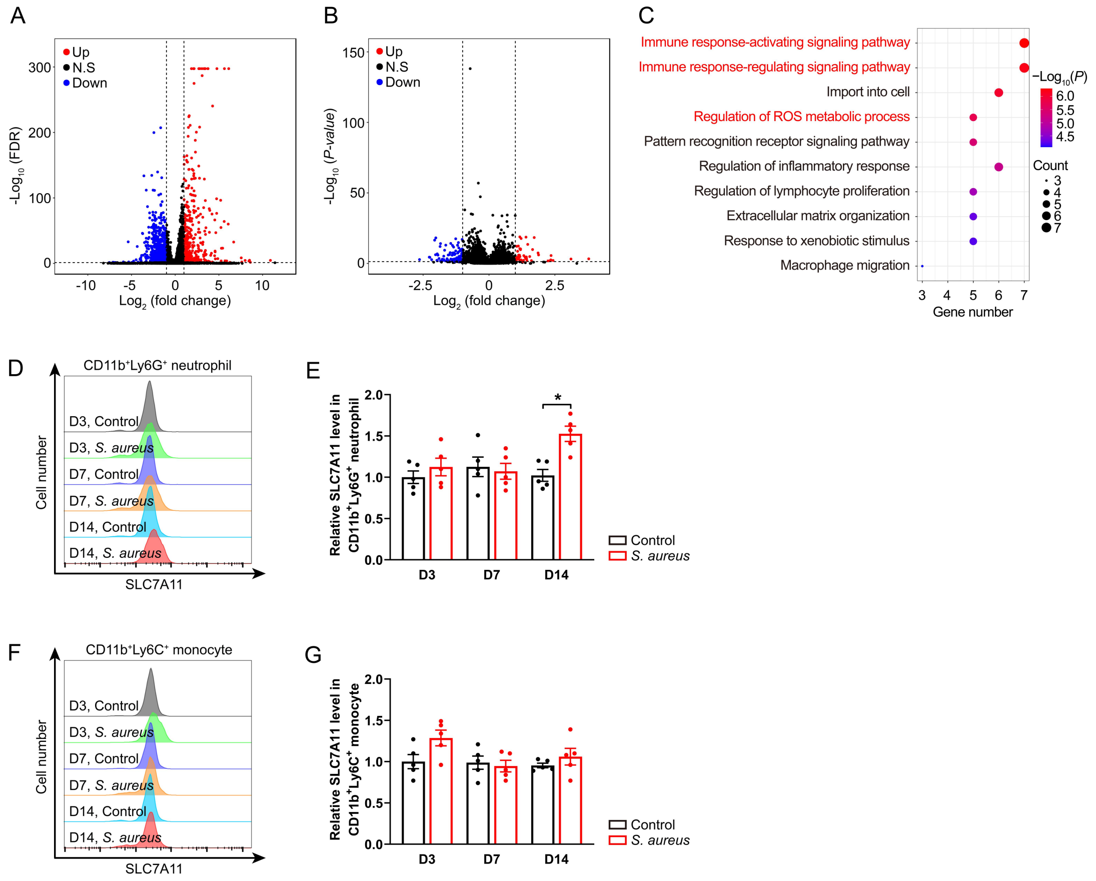

Figure S2. SLC7A11 expression is up-regulated in macrophages after *S. aureus* infection. (A and B) Volcano plots of the DEGs in the transcriptomes and translatomes of CD11b^{+}F4/80^{+} cells from the femurs of *S. aureus* osteomyelitis mice and control ones by day 14 after surgery. The red dots indicate genes or proteins with significantly up-regulated expression (Up), the blue dots indicate genes or proteins with significantly down-regulated expression (Down), and the black dots indicate genes or proteins with non-significant differential expression (N.S). **(C)** GO analysis of up-regulated DEGs in both the transcriptomes and translatomes. **(D and E)** Representative images of flow cytometry and quantification of SLC7A11 levels in CD11b^{+}Ly6G^{+} neutrophils from *S. aureus* osteomyelitis mice and control ones. D3, D7, and D14 represent time points of sample collection by days 3, 7, and 14 after surgery, respectively. n = 5/group. **(F and G)** Representative images of flow cytometry and quantification of SLC7A11 levels in CD11b^{+}Ly6C^{+} monocytes from *S. aureus* osteomyelitis mice and control ones. D3, D7, and D14 represent time points of

sample collection by days 3, 7, and 14 after surgery, respectively. n = 5/group. Data are shown as means ± SEM. One-way ANOVA with Fisher's LSD *post hoc* test was used. *$P < 0.05$, **$P < 0.01$, ***$P < 0.001$.

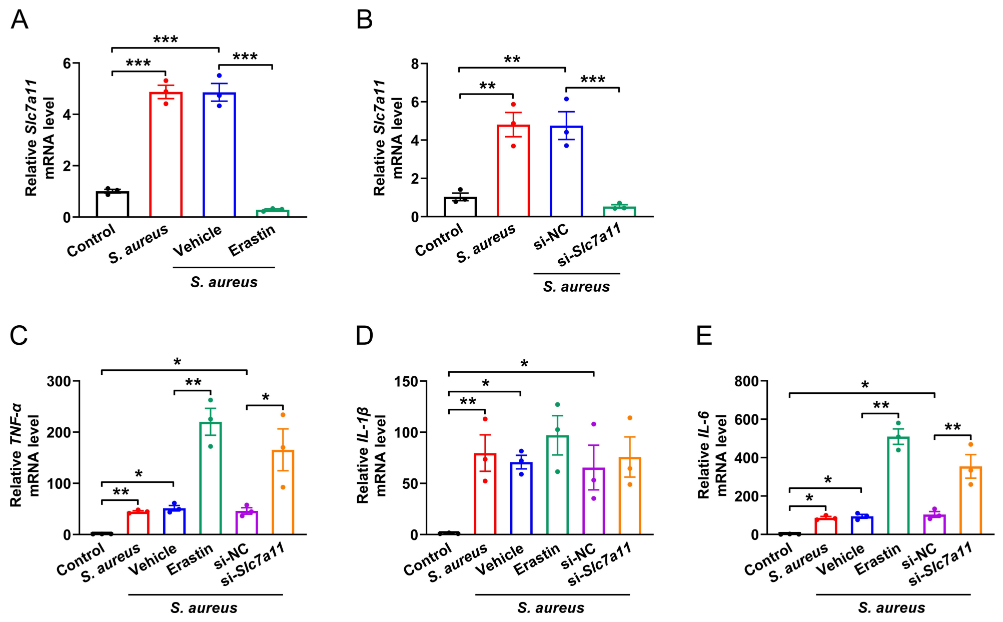

Figure S3. Inhibition of SLC7A11 enhances the bactericidal capacity of macrophages by inducing ROS generation and lipid peroxidation. (A and B) The mRNA expression level of *Slc7a11* in BMDMs after 12 hours of *S. aureus* infection in the presence of erastin (5 μM) or si-*Slc7a11* transfection (100 nM). n = 3/group. **(C-E)** The mRNA expression levels of *TNF-a*, *IL-1β*, and *IL-6* in BMDMs after 12 hours of *S. aureus* infection in the presence of erastin (5 μM) or si-*Slc7a11* transfection (100 nM). n = 3/group. Data are shown as means ± SEM. One-way ANOVA with Fisher's LSD *post hoc* test (A, B, D) or Dunnett's T3 *post hoc* test (C, E) was used. *$P < 0.05$, **$P < 0.01$, ***$P < 0.001$.

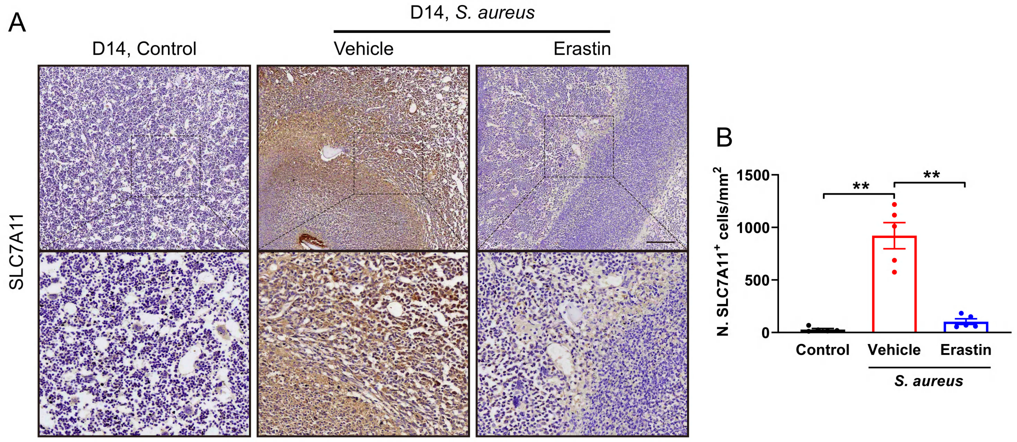

Figure S4. Erastin treatment ameliorates the pathogenesis of *S. aureus* osteomyelitis in mice. Representative immunohistochemical images of SLC7A11 in the femurs from *S. aureus* osteomyelitis mice treated with vehicle or erastin and control ones (A). Quantification of the number of SLC7A11$^+$ cells per mm^2 tissue area (N. SLC7A11$^+$ cells) is shown in (B). Mice were euthanized and the right femurs were collected by day 14 after implant-associated *S. aureus* osteomyelitis surgery. Scale bar, 250 µm. n = 5/group. Data are shown as means ± SEM. One-way ANOVA with Dunnett's T3 *post hoc* test was used. *$P < 0.05$, **$P < 0.01$, ***$P < 0.001$.

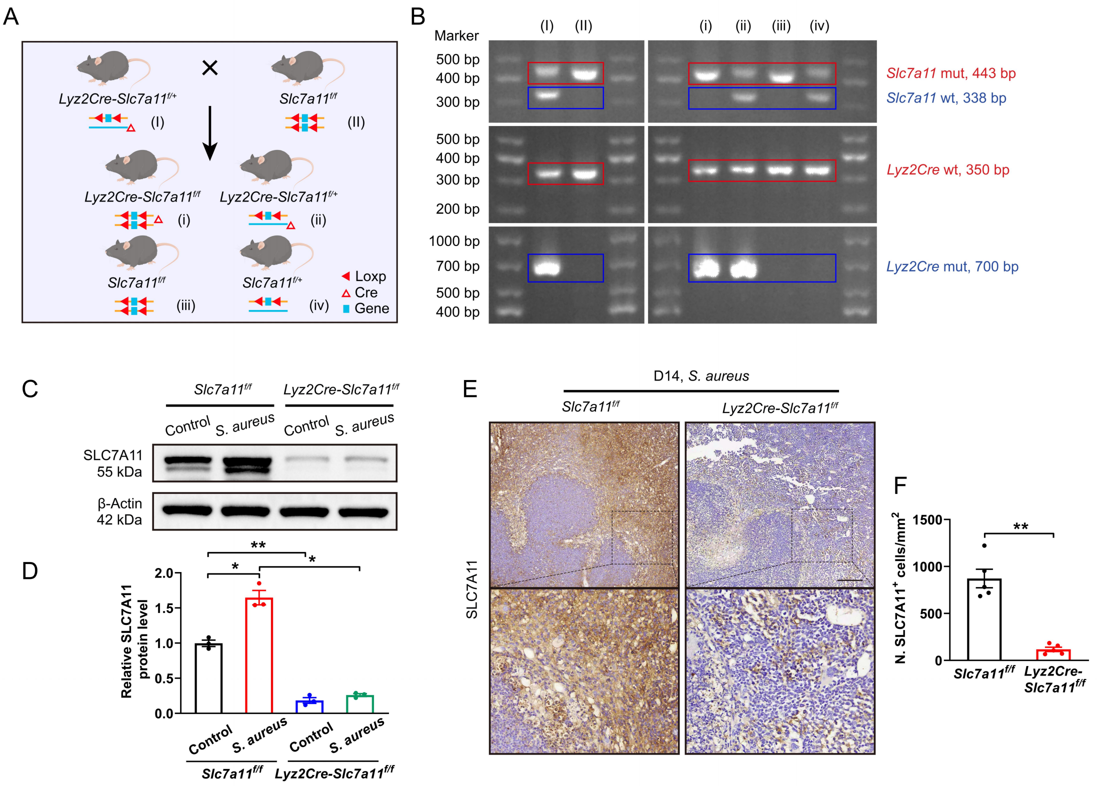

Figure S5. Macrophage-specific knockout of *Slc7a11* ameliorates the pathogenesis of *S. aureus* osteomyelitis in mice. (A) A schematic diagram of the breeding strategy for *Lyz2Cre-Slc7a11$^{f/f}$* mice and *Slc7a11$^{f/f}$* mice. **(B)** Representative images of mouse genotype identification. **(C and D)** Representative images and quantification of western blots for SLC7A11 in BMDMs that were isolated from *Slc7a11$^{f/f}$* mice and *Lyz2Cre-Slc7a11$^{f/f}$* mice and treated with *S. aureus* (MOI = 10) or an equal volume of PBS for 12 hours. n = 3/group. **(E and F)** Representative immunohistochemical images of SLC7A11 in the femurs from *Lyz2Cre-Slc7a11$^{f/f}$* mice and *Slc7a11$^{f/f}$* mice by day 14 after surgery. Quantification of the number of SLC7A11^{+} cells per mm^2 tissue area (N. SLC7A11^{+} cells) is shown in (F). Scale bar, 250 μm. n = 5/group. Data are shown as means ± SEM. Two-way ANOVA with Dunnett's T3 *post hoc* test (D) or unpaired two-tailed Student's *t*-test (F) was used. *P < 0.05, **P < 0.01, ***P < 0.001.

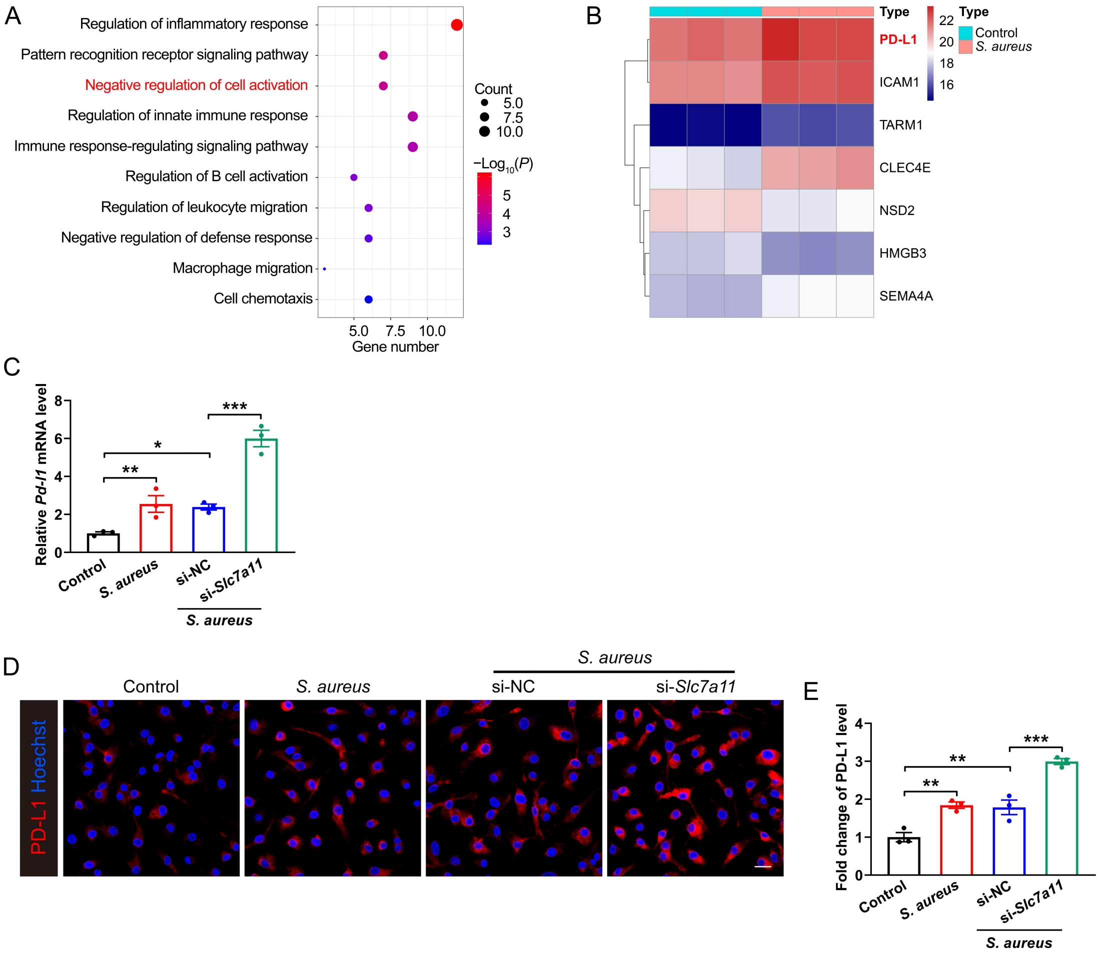

Figure S6. Blocking SLC7A11 promotes PD-L1 expression via the ROS-NF-κB axis in macrophages after *S. aureus* infection. (A) GO analysis of DEGs in the translatomes of CD11b^{+}F4/80^{+} cells from femurs of *S. aureus* infected mice and control ones. GO items with an adjusted *P-value* < 0.05 were considered significantly enriched. **(B)** Heatmap of the DEGs in the GO item "negative regulation of cell activation". **(C)** The mRNA expression level of *Slc7a11* in BMDMs after 12 hours of *S. aureus* infection, with or without si-*Slc7a11* transfection. After 48 hours of transfection with siRNA fragments targeting *Slc7a11* (si-*Slc7a11*) or negative control ones (si-NC), BMDMs were treated with *S. aureus* at a MOI of 10 or an equal volume of PBS. n = 3/group. **(D and E)** Representative images and quantification of immunofluorescence staining for PD-L1 in BMDMs after 12 hours of *S. aureus* infection, with or without si-*Slc7a11* transfection. Scale bar, 20 μm. n = 3/group. Data

are shown as means ± SEM. One-way ANOVA with Fisher's LSD *post hoc* test was used. *$P < 0.05$, **$P < 0.01$, ***$P < 0.001$.
